# A Botanical Mixture Consisting of *Inula japonica* and *Potentilla chinensis* Relieves Obesity via the AMPK Signaling Pathway in 3T3-L1 Adipocytes and HFD-Fed Obese Mice

**DOI:** 10.3390/nu14183685

**Published:** 2022-09-06

**Authors:** Su-Yeon Lee, Kyung-Sook Chung, So-Ri Son, So Young Lee, Dae Sik Jang, Jong-Kil Lee, Hyun-Jae Kim, Chang-Seon Na, Sun-Hee Lee, Kyung-Tae Lee

**Affiliations:** 1 Department of Pharmaceutical Biochemistry, College of Pharmacy, Kyung Hee University, Seoul 02447, Korea; 2 Department of Biomedical and Pharmaceutical Sciences, Graduate School, Kyung Hee University, Seoul 02447, Korea; 3 Department of Pharmacy, College of Pharmacy, Kyung Hee University, Seoul 02447, Korea; 4 Department of New Material Development, COSMAXBIO, Seongnam 13486, Korea

**Keywords:** *Inula japonica*, *Potentilla chinensis*, high-fat diet, AMPK, microbiota

## Abstract

Excessive lipid accumulation in white adipose tissue (WAT) is the major cause of obesity. Herein, we investigated the anti-obesity effect and molecular mechanism of a botanical mixture of 30% EtOH extract from the leaves of *Inula japonica* and *Potentilla chinensis* (EEIP) in 3T3-L1 preadipocytes and high-fat diet (HFD)-fed obese mice. In vitro, EEIP prevented lipid accumulation by downregulating the expression of lipogenesis-related transcription factors such as CCAAT/enhancer binding protein (C/EBP)α, peroxisome proliferator-activated receptor (PPAR)γ, and sterol regulatory element binding protein (SREBP)-1 via AMP-activated protein kinase (AMPK) activation and G_0_/G_1_ cell cycle arrest by regulating the Akt-mTOR pathways without inducing cytotoxicity. In vivo, EEIP significantly reduced body weight gain and body fat mass in the group administered concurrently with HFD (pre-) or administered during the maintenance of HFD (post-) including subcutaneous, gonadal, renal, and mesenteric fats, and improved blood lipid profiles and metabolic hormones. EEIP pre-administration also alleviated WAT hypertrophy and liver lipid accumulation by reducing C/EBPα, PPARγ, and SREBP-1 expression via AMPK activation. In the brown adipose tissue, EEIP pre-administration upregulated the expression of thermogenic factors. Furthermore, EEIP improved the HFD-induced altered gut microbiota in mice. Taken together, our data indicated that EEIP improves HFD-induced obesity through adipogenesis inhibition in the WAT and liver and is a promising dietary natural material for improving obesity.

## 1. Introduction

Modern lifestyles and diets are the leading cause of obesity, and the World Health Organization (WHO) estimates that there are approximately 100 million overweight adults worldwide, 30% of whom are obese [1]. The recent COVID-19 pandemic has limited people’s range of life and has changed their eating habits, which has led to obesity and dyslipidemia [2]. Since obesity is caused by the accumulation of lipids in the body and the increase and expansion of adipose tissue, inhibiting the proliferation and hypertrophy of adipose tissue can help to prevent and treat obesity [3,4]. Adipocyte proliferation is activated through the AMP-activated protein kinase (AMPK) pathway, which regulates adipogenic transcription factors such as peroxisome proliferator-activated γ (PPARγ), CCAT-enhancer binding protein (C/EBPα), and sterol regulatory element-binding protein (SREBP-1) [5]. C/EBP expression is predominant in the adipocytes, hepatocytes, and monocytes/macrophages [6]. PPARs are a type of protein involved in fatty acid oxidation and energy metabolism [7]. Among the three subtypes of PPAR, PPARγ, a member of the nuclear receptor superfamily, is involved in energy balance and the regulation of lipid and glucose homeostasis [8].

In addition, the phosphatidylinositol-3-kinase (PI3K)-protein kinase B (Akt)-mammalian target of the rapamycin (mTOR) pathway, which is initiated by the interaction of PI3K with either G-protein coupled receptors (GPCRs) or receptor tyrosine kinases, is the central regulator of the lipid metabolism involving lipogenesis and lipolysis [9]. Thus, anti-obesity effects can be achieved by regulating this pathway as it plays various roles in adipocyte proliferation and survival [10].

Recent studies have shown that gut microbiomes are strongly linked to obesity [11,12]. Up to 10^14^ gut microbiomes are abundant, and their diversity is known to change depending on the diet and lifestyle [13]. They are responsible for the digestion of carbohydrates and plant polysaccharides into short-chain fatty acids (SCFAs) such as butyrate and acetate. ‘SCFAs, the main metabolites produced by the microbiota, are known to have several beneficial effects on energy metabolism in mammals [14]. Among the microbiome phyla, *Bacteroidetes* and *Firmicutes* account for approximately 90% of the gut and the *Firmicutes*/*Bacteroides* (*F*/*B*) ratio has a pivotal role in maintaining the ideal gut environment [15]. Therefore, changes in the bacterial amount leads to shifts in the *F*/*B* ratio related to various pathological conditions [11].

Although anti-obesity drugs have been long developed, they have limitations with several side effects [16]. For example, orlistat, which is effective in the inhibition of fat digestion and absorption by covalently bonding to the lipase active site [17], has side effects such as steatorrhea and abdominal gas generation. In addition, sibutramine, which induces a reduction of food intake and the formation of satiety, was used as a treatment for obesity but was discontinued after 2010 due to the development of cardiovascular disease [18]. Therefore, there is a need for identifying new natural products for the safe treatment of obesity. *Inula japonica* Thunb. Asteraceae has been studied for its anti-inflammatory effect and anti-obesity activity [4,19]. In addition, *Potentilla chinensis* Ser. Rosaceae reduces inflammation, alleviates non-alcoholic fatty liver disease (NAFLD), and possesses detoxifying properties [20,21]. Based on these reports, we investigated new natural products possessing anti-obesity properties by evaluating the efficacy of a mixture of 30% EtOH extracts from the leaves of *I. japonica* and *P. chinensis* (EEIP) on the loss of body weight and demonstrating the inhibitory mechanism of adipogenic progression.

## 2. Materials and Methods

### 2.1. Materials

Dulbecco’s modified Eagle medium (DMEM), fetal bovine serum (FBS), penicillin, and streptomycin were obtained from Life Technologies (New York, NY, USA). 3-Isobutyl-1-methyl-2,6 (1H,3H)-purinedione (IBMX), dexamethasone, insulin, propidium iodide (PI), sodium orthovanadate, sodium fluoride, phenyl-methylsulfonyl fluoride, protease inhibitor cocktail, and oil red O were obtained from Sigma Aldrich Inc. (St Louis, MO, USA). A protein extraction solution (PRO-PREP™) was obtained from iNtRON Biotechnology (Seongnam, Korea). Antibodies of C/EBPα (#8178), AMPK (#2532), p-AMPKα (T172) (#2535), p-Akt (S273) (#9271), mammalian target of rapamycin (mTOR) (#2972), p-mTOR (S2448) (#2971), p27 (#2552), cytochrome c oxidase IV (COX IV) (#4844), and NAD-dependent deacetylase sirtuin 1 (SIRT1) (#2310) were purchased from Cell Signaling Technology Inc. (Danvers, MA, USA). SREBP-1 (PA1-337) antibody was purchased from Thermo Fisher Scientific Inc. (Waltham, MA, USA). PPARγ (sc-7196), protein kinase B (Akt) (sc-8312), p21 (sc-397), cyclin B (sc-245), cyclin-dependent kinase (CDK) 4 (sc-260), CDK6 (sc-7961), uncoupling protein 1 (UCP-1) (sc-6529), peroxisome proliferator-activated receptor gamma coactivator 1α (PGC-1α) (sc-13068) and β-actin (sc-47778) antibodies were purchased from Santa Cruz Biotechnology Inc. (Dallas, TX, USA).

### 2.2. Sample Preparation and Isolation of Major Compounds from I. japonica and P. chinensis Extracts

Leaves of *Inula japonica* Thunb. (IJ) and *Potentilla chinensis* Ser. (PC) were collected from Sunchang-gun, Jeollabuk-do, and they were classified by MiDNA Genome Research Institute (Gunsan, Korea). Samples of the species (COS2008 and COS2009) were deposited at the herbarium of R&I Center, COSMAX BIO, Seongnam, Korea. Washed and dried leaves of IJ and PC were both extracted individually with 30% EtOH (30 times *w*/*v*) at 80 °C for 5 h, followed by evaporation afforded dried extract residues (35 Brix). The extract residues were sterilized for 1 h and each extract residue (IJE and PCE) was combined at a 1:1 ratio based on the solid state. Thereafter, dextrin was added to the combined mixture and homogenized so that the solid content was at 10% *w*/*w*, then the combined mixture was processed through the spray dryer to obtain a botanical preparation consisting of 30% EtOH extracts of IJ (IJE) and PC extract (PCE) complex (EEIP). IJE (200.0 g) was fractionated by silica gel column chromatography (CC, 70–230 mesh, *ϕ* 8.0 × 55.0 cm, Merck, Darmstadt, Germany) eluting with a gradient solvent system of methylene chloride:MeOH:H_2_O (from 1:0:0 to 3:6.3:0.7, *v*/*v*) to obtain 12 fractions (F1~F12). F10 was separated by Sephadex LH-20 CC (*ϕ* 5.5 × 65.0 cm, MeOH:H_2_O = 7:3, *v*/*v*, Merck) to afford five fractions (F10-1~F10-5). 2,3,4,5-Tetracaffeoyl-d-glucaric acid (2, 50.9 mg) was purified from F10-5 by MCI gel CC (*ϕ* 5.5 × 65.0 cm, Merck) with a gradient solvent system of acetonitrile:0.1% formic acid in H_2_O (from 0:0 to 4:6, *v*/*v*). PCE (100.0 g) was fractionated by Diaion HP-20 CC (*ϕ* 5.5 × 37.0 cm, Merck) with a gradient system of MeOH:water (from 2:8 to 8:2, *v*/*v*) to obtain 13 fractions (R1~R13). Sephadex LH-20 CC (*ϕ* 5 × 57.5 cm, MeOH:H_2_O = 8:2, *v*/*v*) of fraction R6 afforded ten fractions (R6-1~R6-10). Apigenin 7-*O*-*β*-d-glucuronide (1, 51.3 mg) was purified from R6-5 by silica gel CC (230–400 mesh, *ϕ* 3.0 × 32.0 cm) with a gradient solvent system of methylene chloride:MeOH:H_2_O (from 7:3.7:0.3 to 0:9:1, *v*/*v*).

### 2.3. HPLC Analysis for I. japonica and P. chinensis Extracts

In order to standardize the botanical mixture of EEIP, three extracts (IJE, PCE, EEIP) were dissolved in 50% MeOH at a concentration of 50 mg/mL. The standard solutions of apigenin 7-*O*-*β*-d-glucuronide and 2,3,4,5-tetracaffeoyl-d-glucaric acid were prepared in a mixture of DMSO and MeOH (1:9, *v*/*v*) to a concentration of 2000 ppm. Each of the two standard solutions was aliquoted with 500 μL and gently mixed. Finally, the calibration standard mixture was serially diluted and adjusted to the following concentrations: 31.25, 62.5, 125, 250, 500, and 1000 ppm. All samples and calibration solutions were filtered by a 0.2 μm PTFE filter (Whatman Inc., Maidstone, UK). The high-performance liquid chromatogram (HPLC) analysis was conducted by Thermo Fisher Scientific Vanquish™ Horizen Duo UHPLC system (Thermo Fisher Scientific, Sunnyvale, CA, USA), including System Base Vanquish Horizen/Flex, Binary Pump F, Split Samler FT, Column Compartment H, and Diode Array Detecter FG. The analytical column was used by the YMC J’sphere ODS-M80 column (S-4 μm, 8 nm, 250 × 4.6 mm I.D., YMC, Kyoto, Japan). The mobile phases were solvent A (0.1% formic acid in water) and solvent B (0.1% formic acid in acetonitrile) with the following gradient elution: 0 to 7 min, 25 %B; 7 to 37 min, 25 to 65 %B; 37 to 38 min, 65 to 100 %B; 38 to 45 min, 100 %B; 45 to 46 min, 100 to 25 %B; and 46 to 50 min, 25 %B at the flow rate of 0.7 mL/min.

### 2.4. Cell Culture and Cell Viability

The 3T3-L1 cells were purchased from the American Type Culture Collection (ATCC, Manassas, VA, USA). The 3T3-L1 preadipocytes were cultured in growth medium (GM) containing 10% bovine serum (BS) and 1% penicillin–streptomycin (PS) (100 U/mL and 100 μg/mL). The 3T3-L1 preadipocytes were seeded into 96-well plates (2 × 10^5^ cells/well) with growth media (GM) for 24 h at 37 °C in a 5% CO_2_ atmosphere. After 24 h, EEIP was treated through serial dilution and incubated for 1 day. Then, MTT (3-(4,5-Dimethylthizol-2-yl)-2,5- diphenyltetrazolium bromide) solution (5 mg/mL) was treated in each well of 20 uL, then incubated for 4 h at 37 °C. After carefully removing the supernatant, formazan was dissolved in DMSO, and cell viability was measured at 540 nm in a microplate reader (Molecular Devices Inc., San Jose, CA, USA).

### 2.5. Adipocyte Differentiation and Oil Red O Staining for Assessment of Lipid Accumulation

For differentiation, cells were seeded in 6 wells at 2 × 10^5^/mL and cultured confluently for 3 days. After 3 days, cells were completely confluent in wells, changed to differentiation medium (DM) containing MDI (0.5 mM IBMX, 0.5 μM dexamethasone, and 10 μM insulin) treatment, and cultured in DM 10% fetal bovine serum (FBS) and 1% penicillin–streptomycin (PS) and treated with or without EEIP (25, 50, or 100 μg/mL). After 3 days, media were changed to DM with only 1 μM insulin (Sigma-Aldrich), and every 48 h media were changed until 9 days. Finally, the cells are fully differentiated and confluent. Differentiated cells with or without EEIP (25, 50, or 100 μg/mL) treatment in 6 wells for 9 days were treated with oil red O staining for lipid accumulation profiling. Cells were washed by PBS and fixed with 4% formaldehyde for 1 h. Oil red O powder is dissolved in isopropanol at 0.2% and then diluted with DW at a ratio of 40%. After purifying the solution twice with Watman filter paper (diameter 110 μm), staining was performed on the fixed cells for 1 h. Stained cells were observed using an Olympus light microscope system (Tokyo, Japan) after washing with DW. Lipids stained with oil red O were extracted with isopropanol and then measured by a microplate reader (Molecular Devices Inc., San Jose, CA, USA).

### 2.6. Western Blot Analysis

Differentiated cells with or without EEIP (25, 50, or 100 μg/mL) treatment in 6 wells were extracted using PRO-PREP and reacted at room temperature for 30 min to obtain a supernatant at 15,000 rpm at 4 °C. Protein lysates were quantitatively calculated through Bradford assay, and 30 μg of cellular protein was separated by 8–10% polyacrylamide gel electrophoresis (SDS-PAGE) and transferred to the PVDF membrane. At Western blotting, the primary antibody was diluted 1:1000 and reacted over 24 h at 4 °C. Then, the Western blot membrane was washed three times with Tween 20/Tris-buffered saline (T/TBS), and the secondary antibody was diluted at 1:2000 and reacted at 25 °C for 2 h. After washing three times with T/TBS, Amersham hyperfilm ECL (GE Healthcare Life Sciences, Chicago, IL, USA) and ECL chemiluminescent substrate (Santa Cruz Biotechnology Inc., Dallas, TX, USA) was used.

### 2.7. Propidium Iodide (PI) Staining

Preadipocyte cells treated with or without DM + MDI and EEIP were incubated for 24 h, and all were collected. After resuspension in 100 μL PBS, it was added dropwise to 1 mL cold EtOH and fixed overnight at 4 °C. Fixed cells were resuspended in PI staining buffer (100 μg/mL PI staining solution in PBS with 10 μg/mL RNase) and incubated for 20 min in the dark. PI-stained cells were analyzed using flow cytometry (Cytomics FC 500, Beckman Coulter Inc., Brea, CA, USA).

### 2.8. Animals and Experiment Scheme

C57BL/6J mice (male, age six weeks, weight 19–21 g) were purchased from Nara-Biotec (Pyeongtaek, Gyeonggi-do, Korea) and mice were subjected to standard laboratory conditions (light–dark cycle: 12 h, 22 ± 1 °C, humidity 50 ± 10%) for 1 week. Animals were divided into 7 experimental groups (*n* = 8/group): Normal diet control group, HFD group, orlistat group, pre-administration EEIP (100 or 300 mg/kg) group, and post-administration EEIP (100 or 300 mg/kg) group. The ingredient composition of a normal and high-fat diet is shown in Table 1. All groups except the control group were treated with a 30% HFD-fed for 15 weeks. The control group was administered the vehicle for 15 weeks, and the post-administration EEIP group started administration from the 10th week. Animals were orally administrated and body weight was measured every week. All groups were fed normal diet or high-fat diet along with a daily supplement of vehicle, orlistat, or EEIP. After 15 weeks, sacrifice was performed. Subcutaneous fat, mesenteric fat, renal fat, gonadal fat, brown fat, and liver were separated and immediately frozen in liquid nitrogen. All animal experiments were approved by the Animal Experimentation Committee of Kyung Hee University (KHUASP-21-290) and conducted according to the animal guidelines of Kyung Hee University.

### 2.9. Body Composition

Before sacrifice, 3 mice of each group were anesthetized with 0.5 mg/kg ketamine and 5 mg/kg xylazine (i.p.). The body fat mass of each group was measured using a whole-body scanning method, dual energy X-ray absorptiometry (DXA) (InAlyzer, Medikors, Seongnam, Korea). Mice body compositions were displayed as red color (fat tissue), blue color (lean tissue), yellow, or green color (differentiated tissue from lean to fat). Fat in tissue ratio and fat weight were also calculated and expressed in percentages and grams.

### 2.10. Histopathological Analysis

After sacrifice, isolated subcutaneous and liver tissues prepared separately for H&E staining were fixed overnight in 4% formaldehyde. The fixed tissue was sliced into 4 μm and inserted into a paraffin block and stained with Mayer’s hematoxylin and eosin (H&E). The stained slice tissue was measured with an optical microscope (Olympus, Tokyo, Japan). Significance was indicated by averaging the observed diameters of five different adipocytes.

### 2.11. Lipid Profiling

After the experiment, blood was extracted from the veins of anesthetized mice with a 10 U/mL heparin sodium-coated syringe. The extracted blood was mixed well and left at room temperature for 30 min. Samples were then centrifuged at 3500× *g* rpm, 25 °C for 10 min. The separated plasma supernatant was stored at −80 °C. Total cholesterol (T-CHO), triglyceride (TG), plasma levels of low-density lipoproteins (LDL), and high-density lipoproteins (HDL).

### 2.12. Biochemistry Analysis in Plasma

Glutamate oxaloacetate transaminase (GOT), glutamate pyruvate transaminase (GPT), blood urea nitrogen (BUN), leptin, and insulin plasma levels were analyzed using an AU480 chemistry analyzer (Beckman Coulter, Brea, CA, USA) from T&P Bio (Gwangju, Korea).

### 2.13. Microbiome Taxonomic Profiling (MTP)

Stools were collected for each mouse before sacrifice and stored at −80 °C. Each stool was extracted with the QIAamp^®^ Fast DNA Stool Mini Kit (Qiagen, Hilden, Germany), and PCR was performed to amplify the V3–V4 region of bacteria. After purification with the ProNex^®^ Size-Selective Purification System (Promega, Madison, WI, USA), only the V3–V4 region of 16S rRNA was purified on a 1.5% agarose gel and QuantiFluor^®^ One dsDNA System (Promega, Madison, WI, USA) was quantified using. PCR primers and analysis were previous reports described [22]. Products quantified at 2 nM were collected in one tube and analyzed using the Illumina iSeq 100 sequencing system (Illumina, San Diego, CA, USA).

### 2.14. Statistical Analysis

Statistical analysis was performed using GraphPad Prism and all data are means ± standard deviation (SD) in vitro or standard error of the mean (SEM) in vivo. Analysis of variance was also performed by Dunnett’s multiple comparison test. Values of *p* < 0.05 or less were considered statistically significant. ^#^ *p* < 0.05 compared with the GM group or control group and * *p* < 0.05, ** *p* < 0.01, *** *p* < 0.001 compared with the DM + MDI group or the HFD group.

## 3. Results

### 3.1. Identification and Quantification of Apigenin 7-O-β-d-Glucuronide and 2,3,4,5-Tetracaffeoyl-d-Glucaric Acid in the EEIP

**Figure 1:**
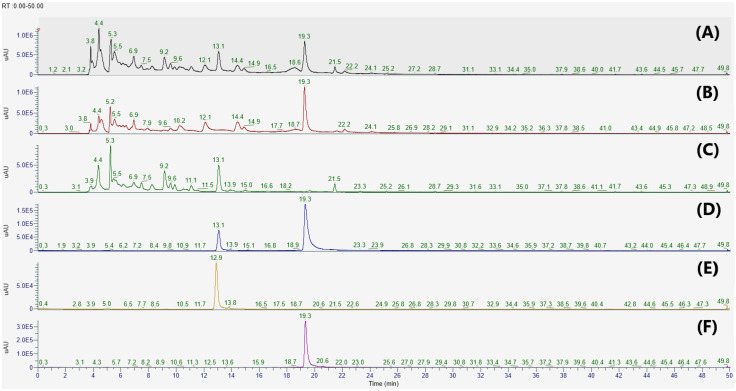
HPLC chromatograms of (**A**) a botanical mixture of 30% EtOH extracts from the leaves of *Inula japonica* and *Potentilla chinensis*, (**B**) 30% EtOH extracts of *I. japonica* Thunb, (**C**) 30% EtOH extracts of *P. chinensis* Ser., and (**D**–**F**) standard solutions at 280 nm. Apigenin 7-*O*-*β*-d-glucuronide and 2,3,4,5-tetracaffeoyl-d-glucaric acid were detected at *R*_t_ 13.1 and 19.3 min, respectively. Calibration standard solutions: (**D**) A mixture of apigenin 7-*O*-*β*-d-glucuronide and 2,3,4,5-tetracaffeoyl-d-glucaric acid, (**E**) apigenin 7-*O*-*β*-d-glucuronide, and (**F**) 2,3,4,5-tetracaffeoyl-d-glucaric acid.

In the present study, apigenin 7-*O*-*β*-d-glucuronide (**1**) and 2,3,4,5-tetracaffeoyl-d-glucaric acid (**2**) were isolated from the PCE and IJE, respectively. The chemical structures of compounds **1** and **2** were identified by comparing their ^1^H-NMR and ESI-MS data to previously published data [23,24]. To determine whether IJE and PCE predominantly contained compounds **1** and **2**, HPLC analyses were performed on each chemical and extract. As shown in Figure 1, compounds **1** and **2** were detected at retention time (*R*_t_) 13.1 and 19.3 min, respectively. These two compounds were observed with strong intensities in each extract of *I*. *japonica* (IJE) and *P*. *chinensis* (PCE), and their presence was also confirmed in a 1:1 mixture of EEIP.

### 3.2. EEIP Regulates Adipogenic Differentiation in 3T3-L1 Preadipocytes

**Figure 2:**
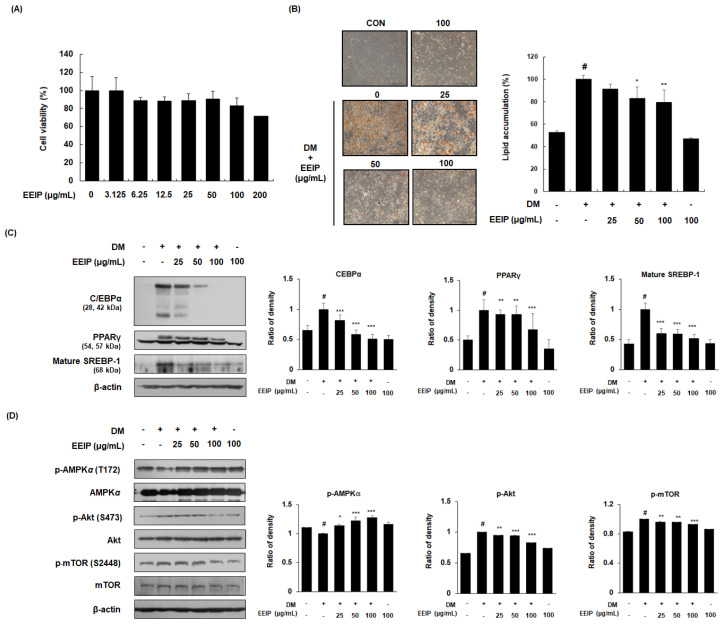
Effect of EEIP on adipocyte differentiation in 3T3−L1 preadipocytes. (**A**) Cell viability was measured using the MTT assay under growth media conditions, with or without EEIP (25, 50, or 100 μg/mL). (**B**) Relative microscopic images and quantitative data of lipid accumulation. For assessment of lipid accumulation, cells were differentiated into adipocytes in DM with or without EEIP (25, 50, or 100 μg/mL) and then oil red O staining was performed. (**C**,**D**) The protein levels of adipogenic transcription factors and the activation of AMPK and the Akt/mTOR pathway in DM−treated cells, with or without EEIP (25, 50, or 100 μg/mL) were analyzed by Western blotting. β-actin was used as the internal control. Densitometric analysis was performed using Bio-Rad Quantity One Software (BioRad; Hercules, CA, USA). Values are represented as the mean ± SD. *^#^ p* < 0.05 vs. the GM group, * *p* < 0.05, ** *p* < 0.01, and *** *p* < 0.001 vs. the DM group.

Prior to analyzing the inhibitory effect of EEIP on adipogenic differentiation, the effect of EEIP on the viability of 3T3-L1 preadipocytes was identified using an MTT assay. As shown in Figure 2A, cell viability was found to be more than 80% when treated with up to 100 μg/mL of EEIP; therefore, further in vitro experiments were performed using 25 to 100 μg/mL EEIP. Differentiation of 3T3-L1 preadipocytes, with or without DM medium containing MDI, resulted in lipid accumulation, whereas EEIP treatment (25, 50, or 100 μg/mL) reduced this accumulation in a concentration-dependent manner (Figure 2B). To further clarify the relationship between adipogenesis-related mediators and AMPK activation in lipid accumulation, the effect of EEIP on the expression of adipogenic transcription markers and AMPK phosphorylation was examined in differentiated 3T3-L1 cells. As shown in Figure 2C, DM treatment markedly increased the protein expression of adipogenic transcription factors including C/EBPα, PPARγ, and mature SREBP-1, while EEIP significantly suppressed these increased expressions in a concentration-dependent manner. In addition, we found that EEIP significantly and concentration-dependently increased the phosphorylation of AMPKα, whereas the total levels of AMPKα were not affected in 3T3-L1 adipocytes (Figure 2D). As Akt and mTOR play key roles in cell proliferation and differentiation [25], we examined the expression of these phosphorylated proteins in DM-induced 3T3-L1 adipocytes, and found that EEIP reduced the DM-induced expression levels of p-Akt/Akt and p-mTOR/mTOR.

### 3.3. EEIP Inhibits Mitotic Clonal Expansion (MCE) by Regulating the Cell Cycle in 3T3-L1 Preadipocytes

**Figure 3:**
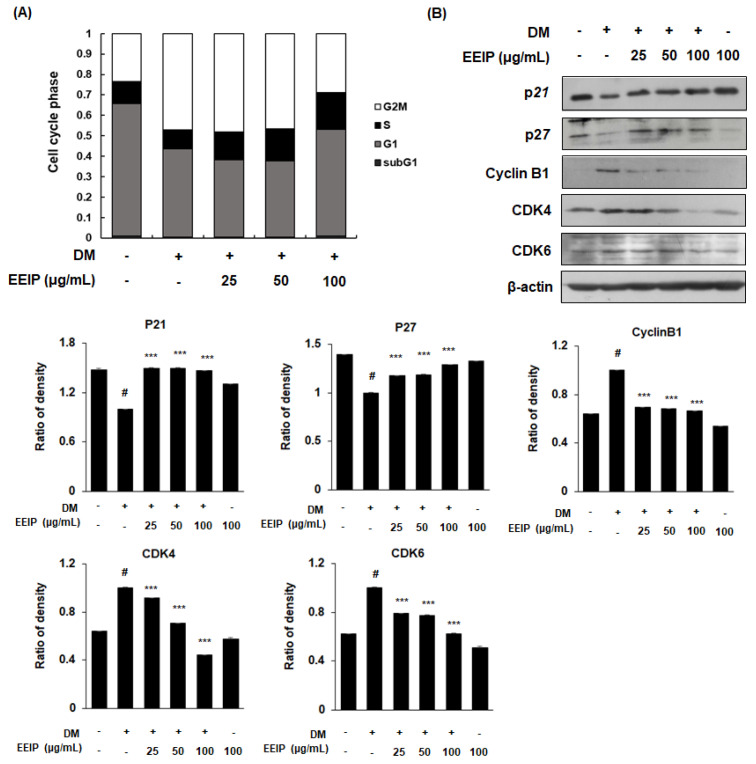
Regulation of mitotic clonal expansion (MCE) in EEIP-treated 3T3-L1 preadipocytes. Cells were differentiated into adipocytes in differentiation media (DM), with or without EEIP (25, 50, or 100 μg/mL), for 24 h. (**A**) Cell cycle progression was analyzed by flow cytometry in EEIP-treated 3T3-L1 preadipocytes. (**B**) Protein levels of cell cycle regulatory proteins were determined by Western blotting analysis. β-actin was used as the internal control. Densitometric analysis was performed using Bio-Rad Quantity One Software (BioRad; Hercules, CA, USA). Values are represented as the mean ± SD, ^#^ *p* < 0.05 vs. the GM group, *** *p* < 0.001 vs. the DM group.

During adipocyte differentiation progression, the adipocytes undergo MCE, which occurs during the early stage of adipogenesis [26]. To investigate the regulation of EEIP on adipocyte proliferation, we analyzed the cell cycle in EEIP-treated 3T3-L1 preadipocytes using flow cytometry. As shown in Figure 3A, the accumulation of the DM-treated cells showed a decreased G_0_/G_1_ phase (from 66.2 to 42.6%), whereas an increased G_2_/M phase (from 23.1% to 48.1%), which indicates the delayed cell cycle arrest in the G_0_/G_1_ phase. In contrast, treatment with 25 and 50 μg/mL EEIP halted the cell accumulation at the S phase (12.6% and 15.0%, respectively) and treatment with 100 μg/mL EEIP recovered up to 55.1% of the G_0_/G_1_ phase. We further examined the effects of EEIP on the cell cycle-related proteins in 3T3-L1 preadipocytes. As expected, our data showed that DM induced the expression of CDK inhibitors (CDKIs), p21 and p27, but decreased that of cyclin B1, CDK4, and CDK6. However, EEIP treatment significantly recovered these DM-induced effects (Figure 3B) suggesting that EEIP reduces adipocyte differentiation by inhibiting MCE through the induction of G_0_/G_1_ phase cell cycle arrest. Consequently, our data suggest that EEIP can inhibit adipogenesis, which leads to lipid accumulation through the regulation of adipogenic transcription factors and cell proliferation-related pathways.

### 3.4. EEIP Relieves Body Weight and Fat Tissue in HFD-Fed Obese Mice

**Figure 4:**
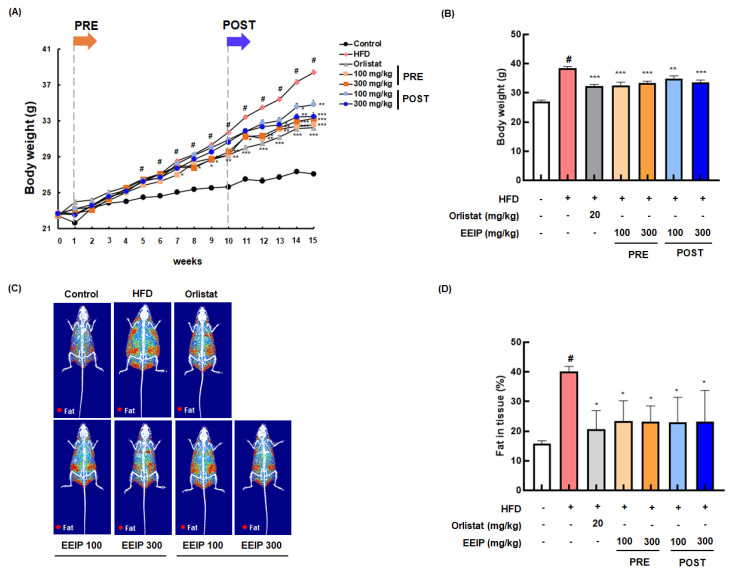

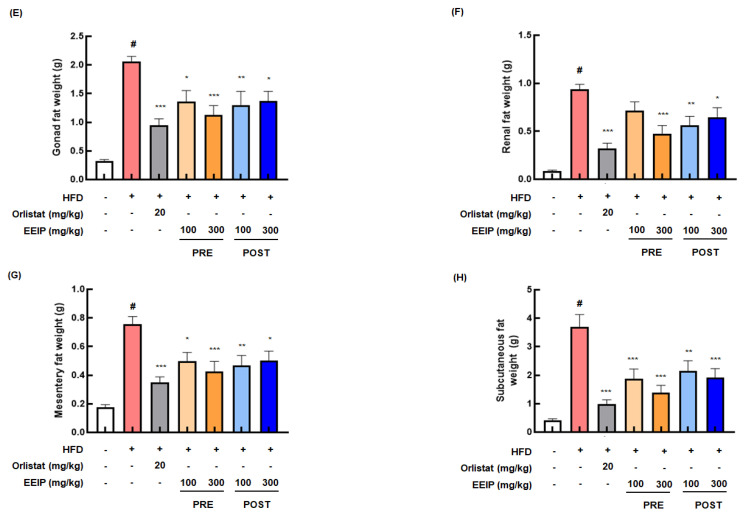
Effect of EEIP on body weight and body fat in HFD-fed obese mice. The pre−administration group was administered EEIP from the 1st week, and the post-administration group was administered EEIP from the 10th week. (**A**) Weekly change in body weight and (**B**) the final body weight. (**C**) The fat distribution, presented with a radiography image, and (**D**) the calculated values of fat in the tissue are measured using DXA. (**E**) Gonad, (**F**) renal, (**G**) mesentery, and (**H**) subcutaneous fat weight. Values are represented as the mean ± SEM. *^#^ p* < 0.05 vs. normal diet control group; * *p* < 0.05, ** *p* < 0.01, and *** *p* < 0.001 vs. HFD group.

To confirm the reducing effect of EEIP on lipid accumulation in vivo, we determined the lowering effect of EEIP, orlistat as a positive control, or vehicle on the body and fat tissue weight in HFD-fed obese mice for 15 weeks. As shown in Figure 4A, the HFD group showed a significant increase in body weight compared to the control group from 5 weeks. In contrast, EEIP pre- and post-administration groups began to show significant body weight gain loss from 7 and 13 weeks, respectively. At the end of the experiment, the body weight of the HFD group was markedly increased compared to that of the control group (27.08 ± 0.24 g at the control group vs. 38.40 ± 0.26 g at the HFD group, *p* < 0.05), and EEIP pre- or post-administration significantly reduced the HFD-increased body weight (38.40 ± 0.26 g at the HFD group vs. 32.56 ± 0.50 g at the EEIP pre-administration 100 mg/kg group, *p* < 0.001; 33.27 ± 0.30 g at the EEIP pre-administration 300 mg/kg group, *p* < 0.001; 34.81 ± 0.45 g at the EEIP post-administration 100 mg/kg group, *p* < 0.01; 33.49 ± 0.45 g at the EEIP post-administration 300 mg/kg group, *p* < 0.01), similar to that observed in the orlistat group (32.24 ± 0.28 g at 20 mg/kg, *p* < 0.001) (Figure 4B). DXA analysis revealed that fat accumulation and fat in the tissue (%) of the HFD group were significantly increased compared with the control group (15.80 ± 0.92% at the control group vs. 40.02 ± 1.82% at the HFD group, *p* < 0.05), whereas in the EEIP pre- or post-administration group, the improvement was similar to that in the orlistat group (40.02 ± 1.82% at the HFD group vs. 23.33 ± 6.90% at the EEIP pre-administration 100 mg/kg group, *p* < 0.01; 23.21 ± 5.18% at the EEIP pre-administration 300 mg/kg group, *p* < 0.01; 22.94 ± 8.46% at the EEIP post-administration 100 mg/kg group, *p* < 0.01; 23.29 ± 10.47% at the EEIP post-administration 300 mg/kg group, *p* < 0.01; 20.56 ± 6.34% at the orlistat 20 mg/kg group, *p* < 0.01, Figure 4C,D). In addition, gonad, renal, mesentery, and subcutaneous fat weight were increased by the HFD, but EEIP pre- or post-administration significantly reduced the weight of each fat (Figure 4E–H), suggesting that EEIP relieves HFD-increased body weight through the suppression of body fat accumulation.

### 3.5. EEIP Improves the Levels of Insulin and Leptin, and the Lipid Profile in the Plasma of HFD-Fed Obese Mice

**Figure 5:**
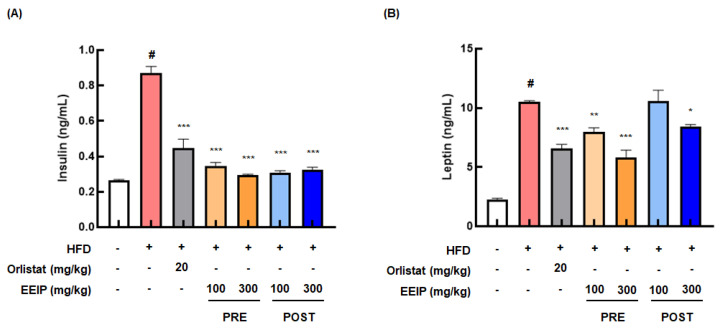
Effect of EEIP on the plasma level of insulin and leptin in HFD−fed obese mice. EEIP modulates plasma levels of (**A**) insulin and (**B**) leptin. Values are represented as the mean ± SEM. *^#^ p* < 0.05 vs. normal diet control group; * *p* < 0.05, ** *p* < 0.01, and *** *p* < 0.001 vs. HFD group.

Since HFD causes hyperlipidemia and cardiovascular disease [27], we analyzed the blood composition, including insulin and leptin levels and lipid profiles in HFD-fed obese mice. As shown in Figure 5A, the HFD group demonstrated remarkably elevated insulin levels (0.26 ± 0.01 ng/mL at the control group vs. 0.86 ± 0.11 ng/mL at the HFD group, *p* < 0.05), while the EEIP pre- or post-administration group showed significantly reduced levels (0.86 ± 0.11 ng/mL at the HFD group vs. 0.33 ± 0.02 ng/mL at the EEIP pre-administration 100 mg/kg group, *p* < 0.001; 0.30 ± 0.02 ng/mL at the EEIP pre-administration 300 mg/kg group, *p* < 0.001; 0.31 ± 0.03 ng/mL at the EEIP post-administration 100 mg/kg group, *p* < 0.001; 0.32 ± 0.04 ng/mL at the EEIP post-administration 300 mg/kg group, *p* < 0.001). Similar to the insulin level, the leptin level was also considerably increased in the HFD group (control group 2.33 ± 0.29 ng/mL vs. HFD group 10.38 ± 0.06 ng/mL, *p* < 0.05), whereas EEIP administration significantly decreased this level except for the post-administration 100 mg/kg group. (10.38 ± 0.06 ng/mL at the HFD group vs. 7.96 ± 1.04 ng/mL at the EEIP pre-administration 100 mg/kg group, *p* < 0.01; 6.10 ± 1.57 ng/mL at the EEIP pre-administration 300 mg/kg group, *p* < 0.001; 8.47 ± 0.44 ng/mL at the EEIP post-administration 300 mg/kg group, *p* < 0.05, Figure 5B).

Among the blood lipids, EEIP pre-administration improved the HFD-induced levels of T-CHO (Table 2, 137.38 ± 16.45 mg/dL at the HFD group vs. 117.87 ± 12.30 mg/dL at the EEIP pre-administration 100 mg/kg group, *p* < 0.05; 116.75 ± 11.03 mg/dL at the EEIP pre-administration 300 mg/kg group, *p* < 0.01) and LDL (11.13 ± 1.96 mg/dL at the HFD group vs. 8.38 ± 1.30 mg/dL at the EEIP pre-administration 100 mg/kg group, *p* < 0.001; 8.88 ± 1.36 mg/dL at the EEIP pre-administration 300 mg/kg group, *p* < 0.01). EEIP toxicity in the liver and kidney was evaluated by measuring the GOT and GPT and BUN levels, respectively. The results showed that GOT, GPT, and BUN levels were not significantly affected by EEIP treatment (Supplementary Figure S1).

### 3.6. EEIP Alleviates Adipogenesis in the Subcutaneous Fat Tissue of HFD-Fed Obese Mice

**Figure 6:**
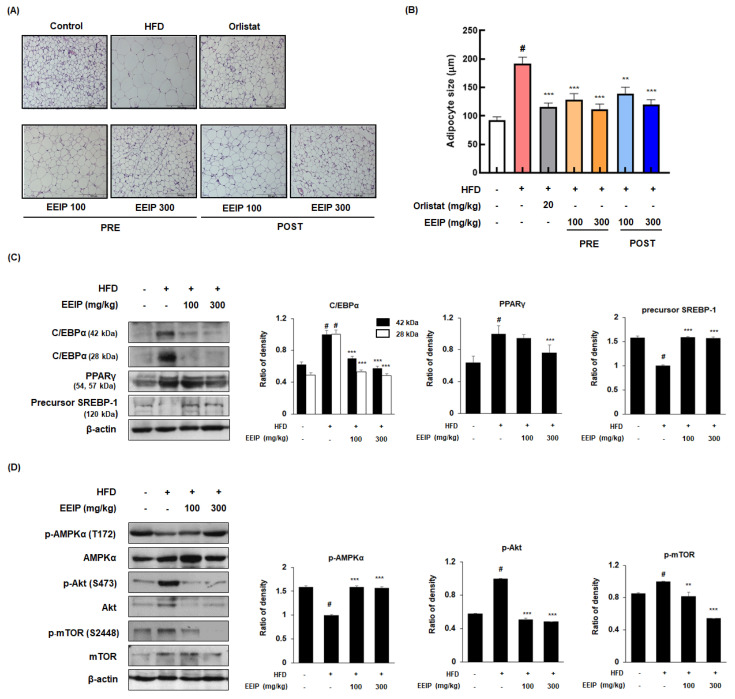
Effects of EEIP on adipocyte size and the levels of adipogenic−related proteins in the subcutaneous fat tissue of HFD−fed obese mice. (**A**) H&E-stained images of subcutaneous adipose tissues. (**B**) The adipocyte diameter was measured through microscopic analysis. (**C**,**D**) Adipogenic−related protein expression was determined by Western blotting analysis and β−actin was used as the internal control. Densitometric analysis was performed using Bio−Rad Quantity One Software (BioRad; Hercules, CA, USA). Values are represented as the mean ± SEM. *^#^ p* < 0.05 vs. normal diet control group; ** *p* < 0.01 and *** *p* < 0.001 vs. HFD group.

As the adipose tissue grows by two processes, hypertrophy and hyperplasia [28], we analyzed the effect of EEIP on the adipocyte hypertrophy of subcutaneous fat using H&E staining. As shown in Figure 6A,B, the HFD group showed an increase in the adipocyte size compared to the control groups (92.28 ± 13.87 μm at the control group vs. 192.2 ± 25.00 μm HFD group, *p* < 0.05); however, EEIP pre- or post-administration decreased the adipocyte size (192.2 ± 25.00 μm at the HFD group vs. 128.4 ± 24.05 μm at EEIP pre-administration 100 mg/kg, *p* < 0.001; 111.8 ± 20.15 μm at EEIP pre-administration 300 mg/kg, *p* < 0.001; 138.6 ± 26.68 μm at the EEIP post-administration 100 mg/kg group, *p* < 0.001; 120.0 ± 19.25 μm at the EEIP post-administration 300 mg/kg group, *p* < 0.001). In addition, Western blotting analysis was performed to clarify the effect of EEIP on the adipogenic transcription factors in the subcutaneous fat. As shown in Figure 6C, EEIP pre-administration restored the changes of C/EBPα and the precursor SREBP-1 protein expression and improved the protein level of PPARγ in subcutaneous fat from HFD-fed obese mice. In addition, both 100 and 300 mg/kg EEIP administration restored the reduced p-AMPK (T172) and elevated p-Akt (S273) and p-mTOR (S2448) levels in the subcutaneous fat of HFD-fed obese mice (Figure 6D), suggesting that EEIP alleviates adipose tissue hypertrophy by regulating adipogenic-related transcription and phosphorylation of the AMPK and Akt/mTOR pathways.

### 3.7. EEIP Prevents Lipid Accumulation and Regulates Adipogenic-Related Protein Expression in Liver Tissue of HFD-Fed Obese Mice

**Figure 7:**
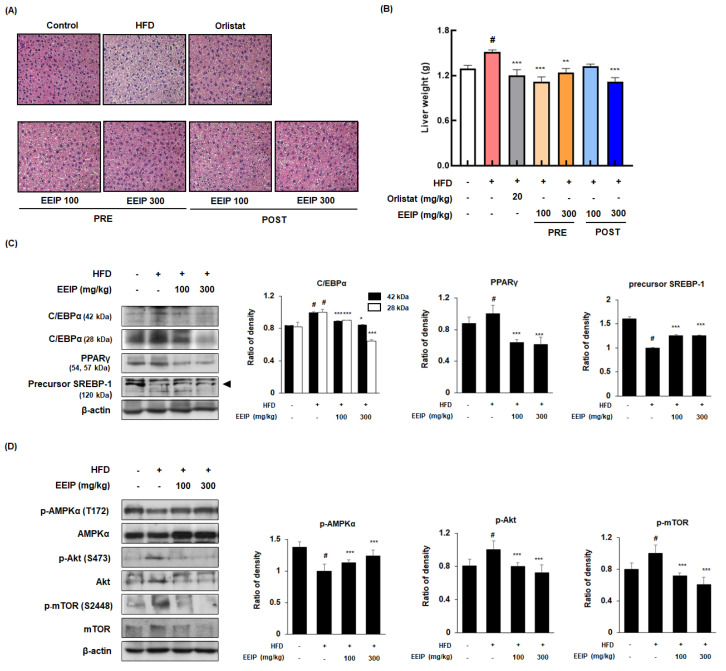
Effect of EEIP on liver fat accumulation in HFD−fed obese mice. (**A**) H&E−stained images from section slides of the liver tissue. (**B**) Assessment of liver weight. (**C**,**D**) Adipogenic−related protein expression of the liver tissue was determined by Western blotting analysis and β-actin was used as the internal control. Densitometric analysis was performed using Bio−Rad Quantity One Software (BioRad; Hercules, CA, USA). Values are represented as the mean ± SEM. *^#^ p* < 0.05 vs. normal diet control group; * *p* < 0.05, ** *p* < 0.01, and *** *p* < 0.001 vs. HFD group.

Since excessive fat accumulation in the liver causes NAFLD [29], we performed H&E staining to analyze the effect of EEIP on lipid accumulation in the liver tissue of HFD-fed obese mice. HFD-treated mice showed increased lipid accumulation with a light-colored image compared to the control mice, whereas EEIP administration alleviated the HFD-induced fat accumulation in the liver tissue (Figure 7A). As shown in Figure 7B, liver weight was significantly increased in the HFD group (1.30 ± 0.12 g at the control group vs. 1.51 ± 0.08 g at the HFD group, *p* < 0.05), while EEIP pre- or post-administration potently restored the liver weight to that of the control level (1.51 ± 0.08 g at the HFD group vs. 1.12 ± 0.16 g at the EEIP pre-administration 100 mg/kg group, *p* < 0.001; 1.24 ± 0.16 g at the EEIP pre-administration 300 mg/kg group, *p* < 0.01; 1.12 ± 0.14 g at the EEIP post-administration 300 mg/kg group, *p* < 0.001). Similar to the results of the subcutaneous fat tissue, HFD-induced expression of the adipogenic transcription factors involving C/EBPα and PPARγ were recovered and SREBP-1 improved by the EEIP administration group in the liver tissue (Figure 7C). Next, we examined the effect of EEIP on the phosphorylation of AMPK, Akt, and mTOR proteins. EEIP administration significantly restored the HFD-fed-activated phosphorylation of AMPK, Akt, and mTOR proteins, suggesting that EEIP also modulates lipid accumulation and adipogenesis in the liver (Figure 7D).

### 3.8. EEIP Stimulates Thermogenesis in the Brown Adipose Tissue of HFD-Fed Obese Mice

**Figure 8:**
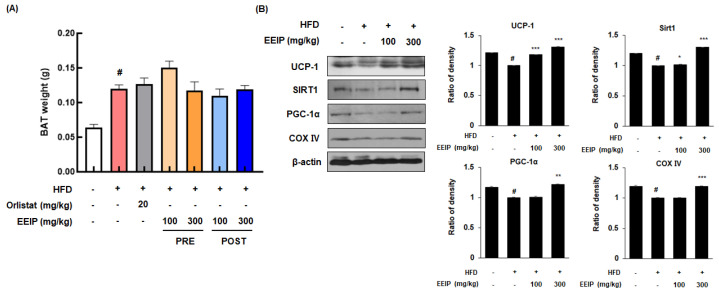
Effect of EEIP pre−administration on thermogenic factors in the brown adipose tissue (BAT) of HFD−fed obese mice (**A**) The brown adipose tissue weight of HFD−fed obese mice. (**B**) The protein expression levels of thermogenesis factors involving UCP−1, SIRT1, PGC−1α, and COX IV were determined by Western blotting analysis and β−actin was used as an internal control. Densitometric analysis was performed using Bio−Rad Quantity One Software (BioRad; Hercules, CA, USA). Values are represented as the mean ± SEM. *^#^ p* < 0.05 vs. normal diet control group; * *p* < 0.05, ** *p* < 0.01, and *** *p* < 0.001 vs. HFD group.

BAT is associated with energy expenditure through uncoupled respiration with high contents of mitochondria and heat production (thermogenesis) [30]. Although there was no significant change in the weight of BAT following EEIP treatment (Figure 8A), the protein expression of major energy consumption factors such as UCP-1, SIRT1, and PGC-1α in BAT was decreased in the HFD-fed mice, while EEIP administration significantly restored these protein expressions, similar to those observed in the control group, except for the EEIP 100 mg/kg group in PGC-1α (Figure 8B). Interestingly, COX IV, which plays a role in ATP synthesis, leading to the triggering of energy metabolism in mitochondria [31], was downregulated by HFD while EEIP administration at 300 mg/kg significantly ameliorated this reduction. These results indicate that EEIP dissipates amounts of chemical energy via an increase in mitochondrial activity of the BAT.

### 3.9. EEIP Restores Gut Microbiome in HFD-Fed Obese Mice

**Figure 9:**
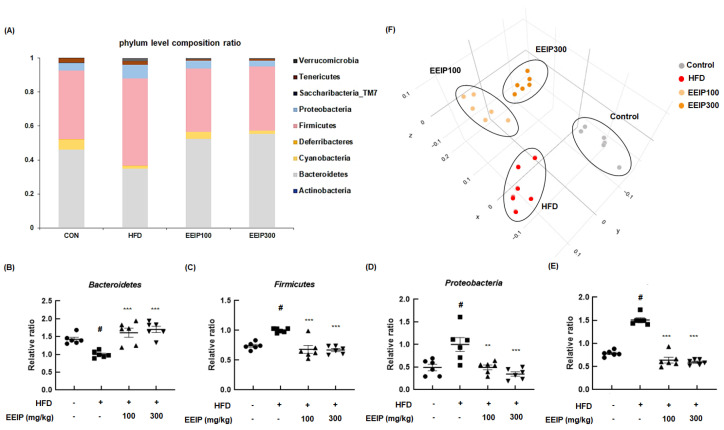
Effect of EEIP pre−administration on gut dysbiosis in HFD−fed obese mice (**A**) phyla level composition ratio (**F**) PCoA graph showing β diversity between groups. (**B**) *Bacteriodetes*, (**C**) *Firmicutes*, (**D**) *Proteobacteria,* and (**E**) *Firmicutes*/*Bacteriodetes* level quantifying graph. Values are represented as the mean ± SEM. *^#^ p* < 0.05 vs. normal diet control group; ** *p* < 0.01, and *** *p* < 0.001 vs. HFD group.

The altered composition of the gut microbiome is closely related to metabolic disorders such as obesity [32]. At the bacterial phyla level, the relative ratio of *Bacteroidetes* was significantly decreased in the HFD group compared to the control group (1.43 ± 0.14 at the control group vs. 1.00 ± 0.09 at the HFD group, *p* < 0.05), while EEIP pre-treatment at 100 and 300 mg/kg significantly recovered the ratio to that of the control group (1.00 ± 0.09 at the HFD group vs. 1.61 ± 0.31 at the EEIP pre-administration 100 mg/kg group, *** *p* < 0.001; 1.70 ± 0.22 at the EEIP pre-administration 300 mg/kg group, *p* < 0.001, Figure 9A,B). In addition, the HFD-induced ratio of *Firmicutes* (1.00 ± 0.03, *p* < 0.05) and *Proteobacteria* (1.00 ± 0.37, *p* < 0.05) was restored by EEIP pre-administration at 100 mg/kg (0.68 ± 0.16, *p* < 0.001 and 0.49 ± 0.13, *p* < 0.01, respectively) and 300 mg/kg (0.67 ± 0.06, *p* < 0.001 and 0.35 ± 0.12, *p* < 0.001, respectively, Figure 9C,D). The relative ratio of *F*/*B* increased rapidly to 1.58 in the HFD group compared to the control group at 0.78, whereas the HFD-induced *F*/*B* ratio was recovered by EEIP administration (0.63 at EEIP pre-administration 100 mg/kg and 0.59 at EEIP pre-administration 300 mg/kg, respectively, Figure 9E). In principal coordinates analysis (PCoA) analysis for β-diversity, we found that there was a difference in diversity between each group and the cluster of EEIP groups has shifted towards the control group (Figure 9F), indicating that EEIP modulates the composition of the gut microbiome in the HFD-fed obese mice to the normal diet control mice.

## 4. Discussion

Modern high-calorie and nutritionally unbalanced diets may have a greater effect on obesity and overweight due to fat accumulation [33]. The incidence of obesity can be reduced by inhibiting the synthesis of fat and/or increasing the body’s energy expenditure [34]. Various natural products have been evaluated for their anti-obesity potential and as a result, they are widely used as an anti-obesity dietary supplement [35]. For example, the green tea extract component catechins have been reported to inhibit lipid absorption and synthesis, relieve fat production, and increase thermogenesis and energy expenditure [36]. In addition, capsaicin, a component of red pepper, has been found to reduce body weight gain and inhibit fat accumulation. As these natural ingredients are stable with relatively low toxicity and have the potential to treat obesity metabolism [37], additional and continuous research on natural products as a treatment for obesity is needed. In the present study, we first evaluated the inhibitory effects of IJE, PCE, and EEIP on TG and lipid accumulation in differentiated-3T3-L1 adipocytes. Interestingly, treatment with EEIP presented stronger amelioration of DM-induced TG levels and lipid accumulation than individual treatment with IJE and PCE in differentiated 3T3-L1 cells (Supplementary Figure S2). Furthermore, it was found that EEIP relieved belly length and body weight without a change in body length in overfeeding (OF)-induced zebrafish (Supplementary Figure S3). Based on these data, EEIP has the potential to be developed as a new functional food candidate for obesity treatment. Therefore, as a part of our ongoing screening program to evaluate the anti-obesity potential of natural compounds, we investigated the effect of EEIP on obesity and its underlying mechanism in vitro and in vivo.

Evidence has highlighted that the occurrence of obesity is related to adipocyte differentiation and maturation [38]. In the early differentiation process, insulin, a differentiation-inducing hormone stimulates C/EBPβ and C/EBPδ to increase the expression of PPARγ. Increased expression of PPARγ promotes CEBPα, which is expressed in the late stage of differentiation, leading to adipocyte proliferation [39]. SREBP, an adipogenic transcription factor present in a membrane-bound inactive form in the ER, affects lipid and cholesterol accumulation by inducing the transcription of genes in the liver and adipose tissue [40]. When SREBP-1 is activated, it is truncated to the mature form, which can bind to sterol response elements in gene promoters to stimulate the transcription of lipogenesis genes such as stearoyl-coenzyme A desaturase 1 and fatty acid synthase, and sterol biosynthesis [41]. Adipocyte maturation includes the induction of MCE and the gene expression of adipogenic transcription factors involved in lipid droplet formation. In the present study, EEIP was found to suppress MCE and regulate the adipogenic transcription factors, including C/EBPα, PPARγ, and SREBP-1, both in vitro and in vivo. It is also well-known that AMPK, a metabolic sensor protein kinase, regulates these adipogenesis-related transcription factors [42] and that the downstream proteins are involved in the maintenance of energy homeostasis in the epididymal adipose tissues [43]. The activity of AMPK inhibits protein synthesis by inhibiting the p70S6 kinase pathway, which is involved in adipocyte hypertrophy. AMPK also decreases the expression of mTOR, the factor that phosphorylates the mTOR-p70S6K pathway and is activated in obesity [44]. Consistent with these reports, it was found that EEIP induced AMPK activation but suppressed the phosphorylation of Akt and mTOR in the adipose and liver tissues. These findings suggest that EEIP-activated AMPK controls the processes of adipogenesis and cell proliferation.

Hepatic lipid accumulation due to excessive caloric intake results in abnormal liver steatosis, which later develops into progressive liver disease [45]. NAFLD is a serious health problem worldwide, with a similar pathological spectrum ranging from simple steatosis to hepatitis, cirrhosis, and hepatocellular carcinoma [46]. In addition, fatty liver due to lipid accumulation is accompanied by metabolic abnormalities such as dyslipidemia, characterized by increased fasting and postprandial TG and LDL levels, and decreased HDL levels [47,48]. In this study, EEIP improved the hepatic lipid constituents including T-CHO, LDL/VLDL, HDL, and TG levels in overfeeding zebrafish (Supplementary Figure S4) and effectively prevented the increased levels of T-CHO and LDL in the plasma of HFD-fed obese mice. Blood levels of GOT and GPT are biomarkers of hepatotoxicity, and BUN level is related to nephrotoxicity. Pre- or post-treatment of EEIP did not show any effects on these levels compared to control mice (Supplementary Figure S1).

Mitochondria-rich BAT is vital for body temperature regulation and contributes to total energy expenditure [49]. Mitochondria provide energy through oxidative phosphorylation of cellular respiration, which proceeds to ATP synthesis through a series of five molecular complexes (complexes I–V). ATP is an end product of mitochondrial respiration and increases the cellular metabolic rate and energy production [50,51]. It has been reported that UCP-1 in the inner mitochondrial membrane of BAT could lead to the dissipation of the proton gradient and the uncoupling respiration to activate thermogenesis rather than ATP synthesis [52]. Increasing evidence suggests that UCP-1 ablation occurs in the development of obesity under thermoneutral conditions [53], and the over-expression of UCP-1 resulting in thermogenesis could prevent the development of obesity [54]. The expression level of UCP-1 is enhanced by an interaction of PGC-1α with thyroid receptors in BAT [55,56]. PGC-1α has been known to express at high levels in tissues where mitochondria are abundant and oxidative metabolism is active, such as BAT. Increased PGC-1α has been known to bind PPAR-γ and stimulate the transcription of genes involved in the brown adipocyte differentiation process [57]. PGC-1α was also found to increase the expression of mitochondrial subunits of electron transport chain complexes such as cytochrome c and COX IV [58]. Further, multiple lines of evidence have demonstrated that SIRT1, an upstream regulator of PGC-1α, ameliorates preadipocyte hyperplasia through c-myc deacetylation and suppresses lipid accumulation by inhibiting PPARγ [59,60]. In line with these reports, heterozygous SIRT1 knockout (SIRT1^+/−^) mice developed severe hepatic steatosis on HFD, accompanied by lower energy consumption [61]. In the present study, Western blotting analysis of BAT demonstrated that EEIP treatment upregulated the expression of energy expenditure-related proteins UCP-1, PGC-1α, and SIRT1. Consistent with these data, EEIP also elevated the expression levels of COX IV, indicating increased mitochondrial activation in BAT of HFD-fed obese mice. Thus, we speculated that EEIP is involved in mitochondrial activity.

Over the past decade, increasing evidence has demonstrated that the gut microbiota is a potential factor in obesity and related metabolic disorders [32]. The gut microbiome mainly comprises *Bacteroidetes*, *Firmicutes*, and *Proteobacteria* at the phyla level, and *Bacteroidetes* and *Firmicutes* account for 90% of this composition. The ratio of *Firmicutes* to *Bacteroidetes* is associated with body mass index and is also an indicator of obesity [62]. In the present study, we observed that the increased *F*/*B* ratio in HFD-induced obese mice was significantly mitigated by EEIP treatment, supporting that EEIP partly alleviates obesity through an alteration of the gut microbiota composition. When the gut microbiome digests dietary fiber, SCFAs such as butyrate, propionate, and acetate are produced as major metabolites [63]. Interestingly, SCFA has been reported to modulate energy metabolism by stimulating leptin production and the regulation of insulin-mediated fat accumulation in the adipose tissue [64,65]. Indeed, our data provide evidence that EEIP restored insulin and leptin levels to those similar to the control in HFD-induced mice, and we hypothesize that EEIP-altered gut microbiota composition might contribute to insulin and leptin secretion via SCFA regulation. In this study, we recognized some limitations of our research to completely unravel the role and molecular mechanism of EEIP in obesity. Although we proved the major compounds of EEIP such as apigenin 7-*O*-*β*-d-glucuronide and 2,3,4,5-tetracaffeoyl-d-glucaric acid in the present study, we anticipated that these compounds could also be responsible for the anti-obesity activities of EEIP. Therefore, we will further investigate on the alleviating effect of 7-*O*-*β*-d-glucuronide and 2,3,4,5-tetracaffeoyl-d-glucaric acid on diet-induced obesity. In addition, further studies are needed to identify whether EEIP exhibits similar effects in human.

## 5. Conclusions

In summary, we elucidated that EEIP inhibited adipocyte proliferation and differentiation and decreased lipid accumulation via the regulation of adipogenesis-related transcription factors by activating the AMPK signaling pathway in vitro and in vivo. In addition, EEIP upregulated the expression of energy expenditure and mitochondrial activation-associated proteins in BAT and restored HFD-altered gut microbiota composition. Taken together, our findings suggest that EEIP possesses the therapeutic potential to prevent obesity and obesity-related metabolic disease.

## Figures and Tables

**Table 1:** Composition of normal and high fat diets.

Proximate Profiles	Normal Diet (%)	High Fat Diet (%)
Protein	18	13
Fat	5.2	30
Crude fiber	6.7	4
Ash	5.7	4
Moisture	4.3	3
Carbohydrate	55.9	41
Others	5.7	4

**Table 2:** Effect of EEIP pre-administration on the plasma level of lipid profile in HFD-fed obese mice.

Variable	Control	HFD	Orlistat (20 mg/kg)	EEIP (100 mg/kg)	EEIP (300 mg/kg)
T-CHO ^a^ (mg/dL)	84.5 ± 7.62	137.38 ± 16.45 ^#^	120.86 ± 12.67 *	117.7 ± 12.30 *	116.75 ± 11.03 **
LDL ^a^ (mg/dL)	7.75 ± 0.89	11.13 ± 1.96 ^#^	8.88 ± 0.99 **	8.38 ± 1.30 ***	8.88 ± 1.36 **
HDL ^a^ (mg/dL)	67.86 ± 3.80	87.71 ± 5.99	92.57 ± 3.65	88.00 ± 3.96	86.71 ± 3.25
TG ^a^ (mg/dL)	52.38 ± 10.21	47.5 ± 10.39	38.63 ± 16.44	36.00 ± 5.78	42.13 ± 9.09

^a^ Values are represented as the mean ± SEM. *^#^ p* < 0.05 vs. normal diet control group; * *p* < 0.05, ** *p* < 0.01, and *** *p* < 0.001 vs. HFD group. T−CHO; Total cholesterol, TG; triglyceride, LDL; low−density lipoproteins, and HDL; high−density lipoproteins.

## Data Availability

Not applicable.

## Supplementary Materials

The following are available online at https://www.mdpi.com/article/10.3390/nu14183685/s1. Figure S1: Assessment of hepatotoxicity and nephrotoxicity of EEIP in the plasma. Figure S2: Effect of IJE, PCE, and EEIP in 3T3-L1. Figure S3: Effect of EEIP treatment group on belly length and body weight in zebrafish. Figure S4: Effect of EEIP on lipid accumulation and T-CHO, LDL/VLDL, HDL, and TG levels in zebrafish.

## References

[1] Levin P.D., Weissman C. (2009). Obesity, metabolic syndrome, and the surgical patient. Med. Clin. N. Am..

[2] Holly J.M.P., Biernacka K., Maskell N., Perks C.M. (2020). Obesity, Diabetes and COVID-19: An Infectious Disease Spreading from the East Collides with the Consequences of an Unhealthy Western Lifestyle. Front. Endocrinol..

[3] Fuster J.J., Ouchi N., Gokce N., Walsh K. (2016). Obesity-Induced Changes in Adipose Tissue Microenvironment and Their Impact on Cardiovascular Disease. Circ. Res..

[4] Park S.H., Lee D.H., Kim M.J., Ahn J., Jang Y.J., Ha T.Y., Jung C.H. (2018). *Inula japonica* Thunb. Flower Ethanol Extract Improves Obesity and Exercise Endurance in Mice Fed a High-Fat Diet. Nutrients.

[5] Ahmad B., Serpell C.J., Fong I.L., Wong E.H. (2020). Molecular Mechanisms of Adipogenesis: The Anti-adipogenic Role of AMP-Activated Protein Kinase. Front. Mol. Biosci..

[6] Nerlov C. (2007). The C/EBP family of transcription factors: A paradigm for interaction between gene expression and proliferation control. Trends Cell Biol..

[7] Hernandez-Quiles M., Broekema M.F., Kalkhoven E. (2021). PPARgamma in Metabolism, Immunity, and Cancer: Unified and Diverse Mechanisms of Action. Front. Endocrinol..

[8] Leonardini A., Laviola L., Perrini S., Natalicchio A., Giorgino F. (2009). Cross-Talk between PPARgamma and Insulin Signaling and Modulation of Insulin Sensitivity. PPAR Res..

[9] Park J.Y., Kang S.E., Ahn K.S., Um J.Y., Yang W.M., Yun M., Lee S.G. (2020). Inhibition of the PI3K-AKT-mTOR pathway suppresses the adipocyte-mediated proliferation and migration of breast cancer cells. J. Cancer.

[10] Huang X., Liu G., Guo J., Su Z. (2018). The PI3K/AKT pathway in obesity and type 2 diabetes. Int. J. Biol. Sci..

[11] Rinninella E., Raoul P., Cintoni M., Franceschi F., Miggiano G.A.D., Gasbarrini A., Mele M.C. (2019). What is the Healthy Gut Microbiota Composition? A Changing Ecosystem across Age, Environment, Diet, and Diseases. Microorganisms.

[12] Hu S., Du M., Su L., Yang H. (2020). Phosphatidylserine from *Portunus trituberculatus* Eggs Alleviates Insulin Resistance and Alters the Gut Microbiota in High-Fat-Diet-Fed Mice. Mar. Drugs.

[13] Davis C.D. (2016). The Gut Microbiome and Its Role in Obesity. Nutr. Today.

[14] den Besten G., van Eunen K., Groen A.K., Venema K., Reijngoud D.J., Bakker B.M. (2013). The role of short-chain fatty acids in the interplay between diet, gut microbiota, and host energy metabolism. J. Lipid Res..

[15] Magne F., Gotteland M., Gauthier L., Zazueta A., Pesoa S., Navarrete P., Balamurugan R. (2020). The Firmicutes/Bacteroidetes Ratio: A Relevant Marker of Gut Dysbiosis in Obese Patients?. Nutrients.

[16] Kang J.G., Park C.Y. (2012). Anti-Obesity Drugs: A Review about Their Effects and Safety. Diabetes Metab. J..

[17] Guerciolini R. (1997). Mode of action of orlistat. Int. J. Obes. Relat. Metab. Disord..

[18] Araujo J.R., Martel F. (2012). Sibutramine effects on central mechanisms regulating energy homeostasis. Curr. Neuropharmacol..

[19] Chen X., Tang S.A., Lee E., Qiu Y., Wang R., Duan H.Q., Dan S., Jin M., Kong D. (2015). IVSE, isolated from *Inula japonica*, suppresses LPS-induced NO production via NF-kappaB and MAPK inactivation in RAW264.7 cells. Life Sci..

[20] Qiao W., Zhao C., Qin N., Zhai H.Y., Duan H.Q. (2011). Identification of trans-tiliroside as active principle with anti-hyperglycemic, anti-hyperlipidemic and antioxidant effects from Potentilla chinesis. J. Ethnopharmacol..

[21] Wang D., Lao L., Pang X., Qiao Q., Pang L., Feng Z., Bai F., Sun X., Lin X., Wei J. (2018). Asiatic acid from *Potentilla chinensis* alleviates non-alcoholic fatty liver by regulating endoplasmic reticulum stress and lipid metabolism. Int. Immunopharmacol..

[22] Han H.S., Lee H.H., Gil H.S., Chung K.S., Kim J.K., Kim D.H., Yoon J., Chung E.K., Lee J.K., Yang W.M. (2021). Standardized hot water extract from the leaves of *Hydrangea serrata* (Thunb.) Ser. alleviates obesity via the AMPK pathway and modulation of the gut microbiota composition in high fat diet-induced obese mice. Food Funct..

[23] Ma Q., Zhang X., Jiang J., Zhu W. (2017). Apigenin-7-O-β-D-glucuronide inhibits modified low-density lipoprotein uptake and foam cell formation in macro-phages. J. Funct. Foods.

[24] Dudek M.K., Dudkowski Ł., Bazylko A., Kaźmierski S., Kiss A.K. (2016). Caffeic acid derivatives isolated from the aerial parts of Galinsoga parviflora and their effect on inhibit-ing oxidative burst in human neutrophils. Phytochem. Lett..

[25] Agathocleous M., Harris W.A. (2013). Metabolism in physiological cell proliferation and differentiation. Trends Cell Biol..

[26] Tang Q.Q., Otto T.C., Lane M.D. (2003). Mitotic clonal expansion: A synchronous process required for adipogenesis. Proc. Natl. Acad. Sci. USA.

[27] Klop B., Elte J.W., Cabezas M.C. (2013). Dyslipidemia in obesity: Mechanisms and potential targets. Nutrients.

[28] Jo J., Gavrilova O., Pack S., Jou W., Mullen S., Sumner A.E., Cushman S.W., Periwal V. (2009). Hypertrophy and/or Hyperplasia: Dynamics of Adipose Tissue Growth. PLoS Comput. Biol..

[29] Grundy S.M. (2000). Metabolic complications of obesity. Endocrine.

[30] Fenzl A., Kiefer F.W. (2014). Brown adipose tissue and thermogenesis. Horm. Mol. Biol. Clin. Investig..

[31] Acin-Perez R., Gatti D.L., Bai Y., Manfredi G. (2011). Protein phosphorylation and prevention of cytochrome oxidase inhibition by ATP: Coupled mechanisms of energy metabolism regulation. Cell Metab..

[32] Muscogiuri G., Cantone E., Cassarano S., Tuccinardi D., Barrea L., Savastano S., Colao A., on behalf of the Obesity Programs of nutrition, Education, Research and Assessment (OPERA) Group (2019). Gut microbiota: A new path to treat obesity. Int. J. Obes. Suppl..

[33] Camacho S., Ruppel A. (2017). Is the calorie concept a real solution to the obesity epidemic?. Glob. Health Action.

[34] Bray G.A., Popkin B.M. (1998). Dietary fat intake does affect obesity!. Am. J. Clin. Nutr..

[35] Sun N.N., Wu T.Y., Chau C.F. (2016). Natural Dietary and Herbal Products in Anti-Obesity Treatment. Molecules.

[36] Huang J., Wang Y., Xie Z., Zhou Y., Zhang Y., Wan X. (2014). The anti-obesity effects of green tea in human intervention and basic molecular studies. Eur. J. Clin. Nutr..

[37] Li R., Lan Y., Chen C., Cao Y., Huang Q., Ho C.T., Lu M. (2020). Anti-obesity effects of capsaicin and the underlying mechanisms: A review. Food Funct..

[38] Munir H., Ward L.S.C., Sheriff L., Kemble S., Nayar S., Barone F., Nash G.B., McGettrick H.M. (2017). Adipogenic Differentiation of Mesenchymal Stem Cells Alters Their Immunomodulatory Properties in a Tissue-Specific Manner. Stem Cells.

[39] Rosen E.D., Hsu C.H., Wang X., Sakai S., Freeman M.W., Gonzalez F.J., Spiegelman B.M. (2002). C/EBPalpha induces adipogenesis through PPARgamma: A unified pathway. Genes Dev..

[40] Crewe C., Zhu Y., Paschoal V.A., Joffin N., Ghaben A.L., Gordillo R., Oh D.Y., Liang G., Horton J.D., Scherer P.E. (2019). SREBP-regulated adipocyte lipogenesis is dependent on substrate availability and redox modulation of mTORC1. JCI Insight.

[41] Hinds T.D., Kipp Z.A., Xu M., Yiannikouris F.B., Morris A.J., Stec D.F., Wahli W., Stec D.E. (2021). Adipose-Specific PPARalpha Knockout Mice Have Increased Lipogenesis by PASK-SREBP1 Signaling and a Polarity Shift to Inflammatory Macrophages in White Adipose Tissue. Cells.

[42] Canto C., Auwerx J. (2009). PGC-1alpha, SIRT1 and AMPK, an energy sensing network that controls energy expenditure. Curr. Opin. Lipidol..

[43] Ix J.H., Sharma K. (2010). Mechanisms linking obesity, chronic kidney disease, and fatty liver disease: The roles of fetuin-A, adiponectin, and AMPK. J. Am. Soc. Nephrol..

[44] Sun X., Han F., Lu Q., Li X., Ren D., Zhang J., Han Y., Xiang Y.K., Li J. (2020). Empagliflozin Ameliorates Obesity-Related Cardiac Dysfunction by Regulating Sestrin2-Mediated AMPK-mTOR Signaling and Redox Homeostasis in High-Fat Diet-Induced Obese Mice. Diabetes.

[45] Liu Q., Bengmark S., Qu S. (2010). The role of hepatic fat accumulation in pathogenesis of non-alcoholic fatty liver disease (NAFLD). Lipids Health Dis..

[46] Singh S., Osna N.A., Kharbanda K.K. (2017). Treatment options for alcoholic and non-alcoholic fatty liver disease: A review. World J. Gastroenterol..

[47] Zhang Q.Q., Lu L.G. (2015). Nonalcoholic Fatty Liver Disease: Dyslipidemia, Risk for Cardiovascular Complications, and Treatment Strategy. J. Clin. Transl. Hepatol..

[48] Wu L., Parhofer K.G. (2014). Diabetic dyslipidemia. Metabolism.

[49] Tsubota A., Okamatsu-Ogura Y., Bariuan J.V., Mae J., Matsuoka S., Nio-Kobayashi J., Kimura K. (2019). Role of brown adipose tissue in body temperature control during the early postnatal period in Syrian hamsters and mice. J. Vet. Med. Sci..

[50] Kobayashi A., Azuma K., Ikeda K., Inoue S. (2020). Mechanisms Underlying the Regulation of Mitochondrial Respiratory Chain Complexes by Nuclear Steroid Receptors. Int. J. Mol. Sci..

[51] Bonora M., Patergnani S., Rimessi A., De Marchi E., Suski J.M., Bononi A., Giorgi C., Marchi S., Missiroli S., Poletti F. (2012). ATP synthesis and storage. Purinergic Signal..

[52] Fedorenko A., Lishko P.V., Kirichok Y. (2012). Mechanism of fatty-acid-dependent UCP1 uncoupling in brown fat mitochondria. Cell.

[53] Feldmann H.M., Golozoubova V., Cannon B., Nedergaard J. (2009). UCP1 ablation induces obesity and abolishes diet-induced thermogenesis in mice exempt from thermal stress by living at thermoneutrality. Cell Metab..

[54] Klaus S., Keipert S., Rossmeisl M., Kopecky J. (2012). Augmenting energy expenditure by mitochondrial uncoupling: A role of AMP-activated protein kinase. Genes Nutr..

[55] Sharma B.K., Patil M., Satyanarayana A. (2014). Negative regulators of brown adipose tissue (BAT)-mediated thermogenesis. J. Cell Physiol..

[56] Liang H., Ward W.F. (2006). PGC-1alpha: A key regulator of energy metabolism. Adv. Physiol. Educ..

[57] Lowell B.B. (1999). PPARgamma: An essential regulator of adipogenesis and modulator of fat cell function. Cell.

[58] Scarpulla R.C. (2002). Transcriptional activators and coactivators in the nuclear control of mitochondrial function in mammalian cells. Gene.

[59] Majeed Y., Halabi N., Madani A.Y., Engelke R., Bhagwat A.M., Abdesselem H., Agha M.V., Vakayil M., Courjaret R., Goswami N. (2021). SIRT1 promotes lipid metabolism and mitochondrial biogenesis in adipocytes and coordinates adipogenesis by targeting key enzymatic pathways. Sci. Rep..

[60] Shen S.H., Singh S.P., Raffaele M., Waldman M., Hochhauser E., Ospino J., Arad M., Peterson S.J. (2022). Adipocyte-Specific Expression of PGC1alpha Promotes Adipocyte Browning and Alleviates Obesity-Induced Metabolic Dysfunction in an HO-1-Dependent Fashion. Antioxidants.

[61] Xu F., Gao Z., Zhang J., Rivera C.A., Yin J., Weng J., Ye J. (2010). Lack of SIRT1 (Mammalian Sirtuin 1) activity leads to liver steatosis in the SIRT1+/− mice: A role of lipid mobilization and inflammation. Endocrinology.

[62] Koliada A., Syzenko G., Moseiko V., Budovska L., Puchkov K., Perederiy V., Gavalko Y., Dorofeyev A., Romanenko M., Tkach S. (2017). Association between body mass index and Firmicutes/Bacteroidetes ratio in an adult Ukrainian population. BMC Microbiol..

[63] Lu Y., Fan C., Li P., Lu Y., Chang X., Qi K. (2016). Short Chain Fatty Acids Prevent High-fat-diet-induced Obesity in Mice by Regulating G Protein-coupled Receptors and Gut Microbiota. Sci. Rep..

[64] Kimura I., Ozawa K., Inoue D., Imamura T., Kimura K., Maeda T., Terasawa K., Kashihara D., Hirano K., Tani T. (2013). The gut microbiota suppresses insulin-mediated fat accumulation via the short-chain fatty acid receptor GPR43. Nat. Commun..

[65] Xiong Y., Miyamoto N., Shibata K., Valasek M.A., Motoike T., Kedzierski R.M., Yanagisawa M. (2004). Short-chain fatty acids stimulate leptin production in adipocytes through the G protein-coupled receptor GPR41. Proc. Natl. Acad. Sci. USA.

