# Supplementary material for: A Botanical Mixture Consisting of Inula japonica and Potentilla chinensis Relieves Obesity via the AMPK Signaling Pathway in 3T3-L1 Adipocytes and HFD-Fed Obese Mice
Source: Nutrients. 2022 Sep 6;14(18):3685. doi: 10.3390/nu14183685 (PMC9505034; doi:10.3390/nu14183685)
Supplement: Supplementary file 1 [file nutrients-14-03685-s001.zip › [Nutrients-1877696]supplementary Data.pdf]

nutrients

MDPI

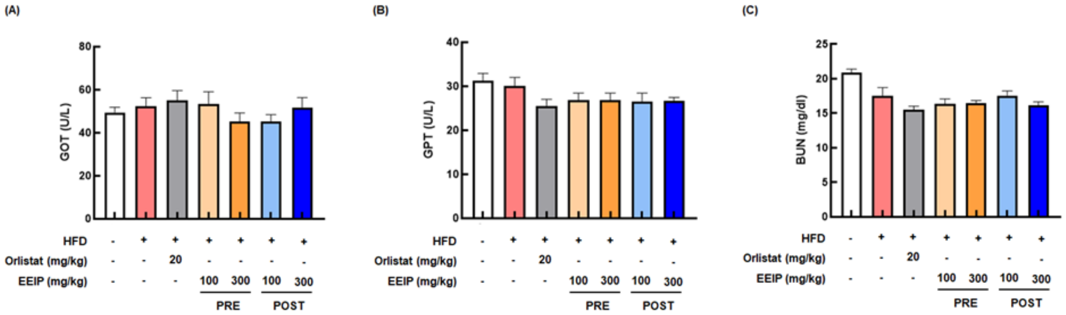

Figure S1. Assessment of hepatotoxicity and nephrotoxicity of EEIP in the plasma. (A) Glutamate oxaloacetate transaminase (GOT), (B) glutamate pyruvate transaminase (GPT), and (C) blood urea nitrogen (BUN) levels.

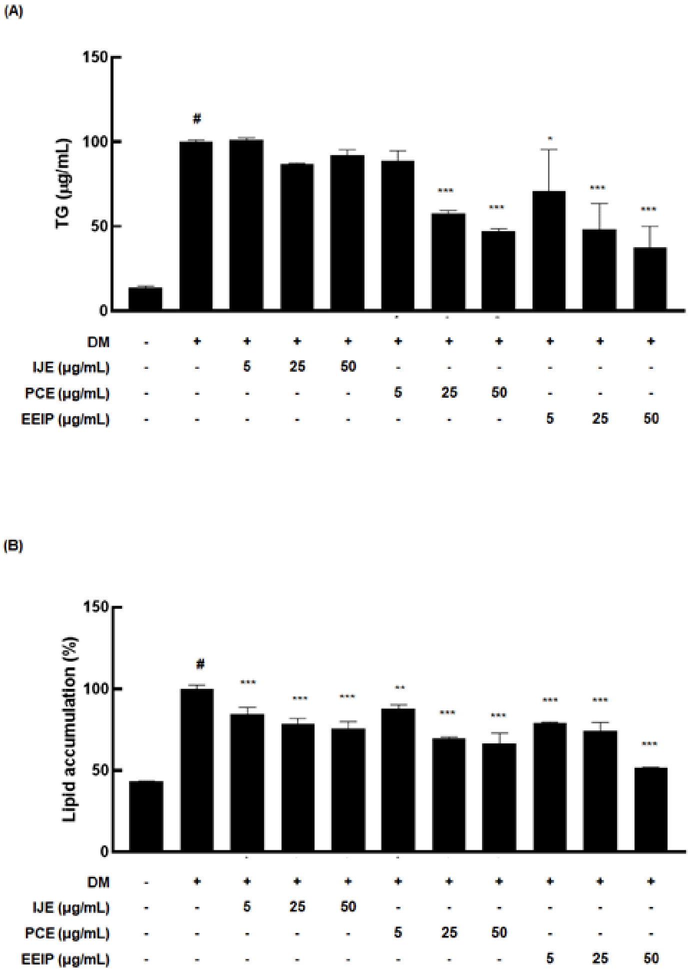

Figure S2. Effect of IJE, PCE, and EEIP in 3T3-L1. Cells were differentiated into adipocytes in differentiation media (DM) with or without IJE (5, 25, and 50 μg/mL), PCE (5, 25, and 50 μg/mL), and EEIP (5, 25, and 50 μg/mL) (A) Alleviation of triglyceride level by IJE, PCE, and EEIP in 3T3-L1 cells. (B) Inhibitory effect of lipid accumulation by IJE, PCE, and EEIP in 3T3-L1 cells. Values are represented as the mean ± SD. $^{\#}p < 0.05$ vs. the GM group, $^{*}p < 0.05$, $^{**}p < 0.01$, $^{***}p < 0.001$ vs. the DM group.

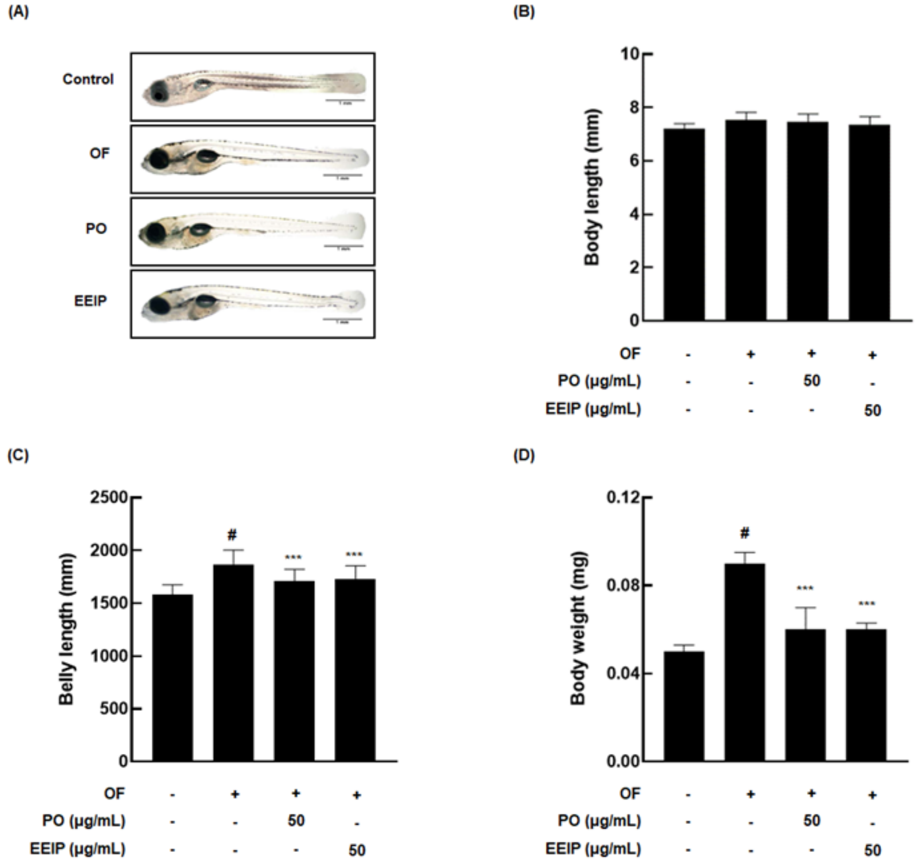

Figure S3. Effect of EEIP treatment group on belly length and body weight in zebrafish. Zebrafish were measured in overfeeding (OF) conditions with or without 50 μg/mL positive control (PO) or EEIP 50 μg/mL. (A, B) Body length in control, OF, PO, and EEIP groups. (C, D) Inhibitory effect of EEIP on belly length and body weight in OF-induced zebrafish. Values as represented as mean ± SD. $^{\#}p < 0.05$ vs. the normal diet control group, $^{*}p < 0.05$, $^{**}p < 0.01$, $^{***}p < 0.001$ vs. the OF group.

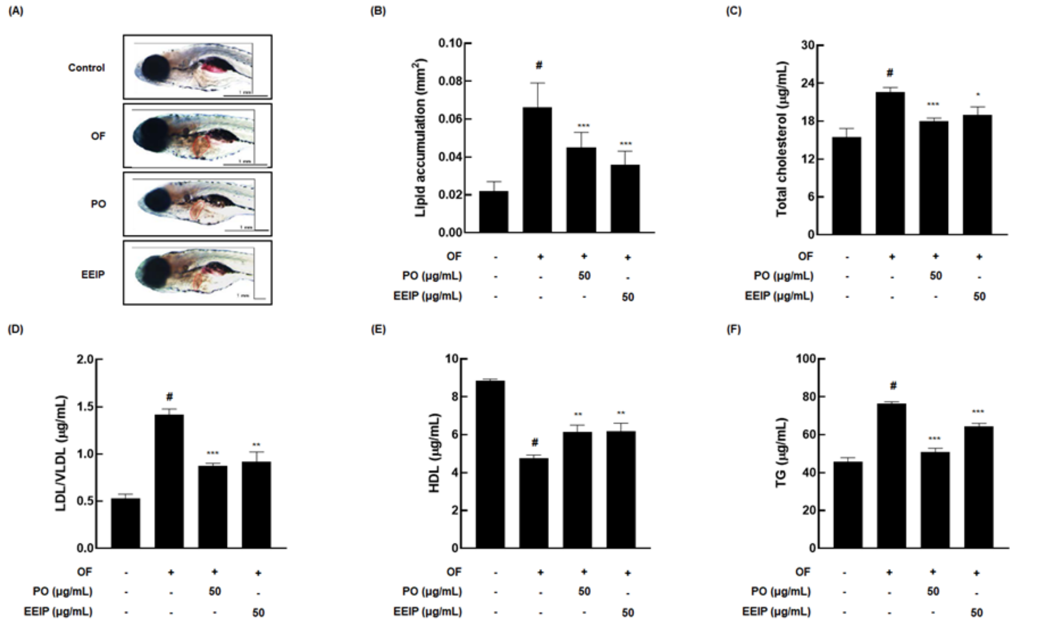

Figure S4. Effect of EEIP on lipid accumulation and T-CHO, LDL/VLDL, HDL, and TG levels in zebrafish. Zebrafish were measured in OF conditions with or without positive control (PO, 50 μg/mL) or EEIP (50 μg/mL). (A, B) Relative microscopic images and quantitative data of lipid accumulation in zebrafish. To evaluate lipid accumulation, zebrafish were induced to obesity in OF conditions with or without PO (50 μg/mL) or EEIP (50 μg/mL), followed by oil Red O staining. Improvement effect of EEIP on (C) T-CHO, (D) LDL/VLDL, (E) HDL, and (F) TG levels. Values are represented as the mean ± SD. $^{\#}p < 0.05$ vs. the normal diet control group, $^{*}p < 0.05$, $^{**}p < 0.01$, $^{***}p < 0.001$ vs. the OF group.
