# Supplementary material for: A Botanical Mixture Consisting of Inula japonica and Potentilla chinensis Relieves Obesity via the AMPK Signaling Pathway in 3T3-L1 Adipocytes and HFD-Fed Obese Mice
Source: Nutrients. 2022 Sep 6;14(18):3685. doi: 10.3390/nu14183685 (PMC9505034; doi:10.3390/nu14183685)

Figure S2A

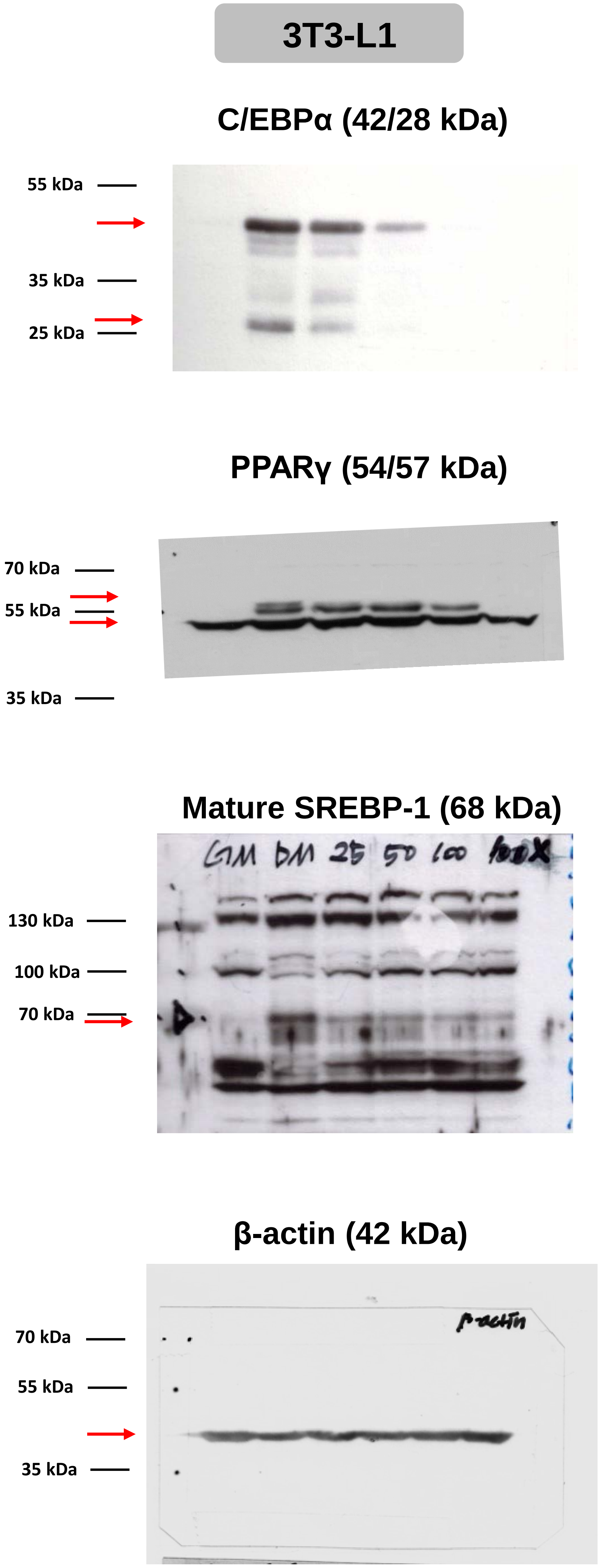
3T3-L1
C/EBPα (42/28 kDa)
55 kDa
35 kDa
25 kDa
PPARγ (54/57 kDa)
70 kDa
55 kDa
35 kDa
Mature SREBP-1 (68 kDa)
130 kDa
100 kDa
70 kDa
β-actin (42 kDa)
70 kDa
55 kDa
35 kDa

Figure S2B

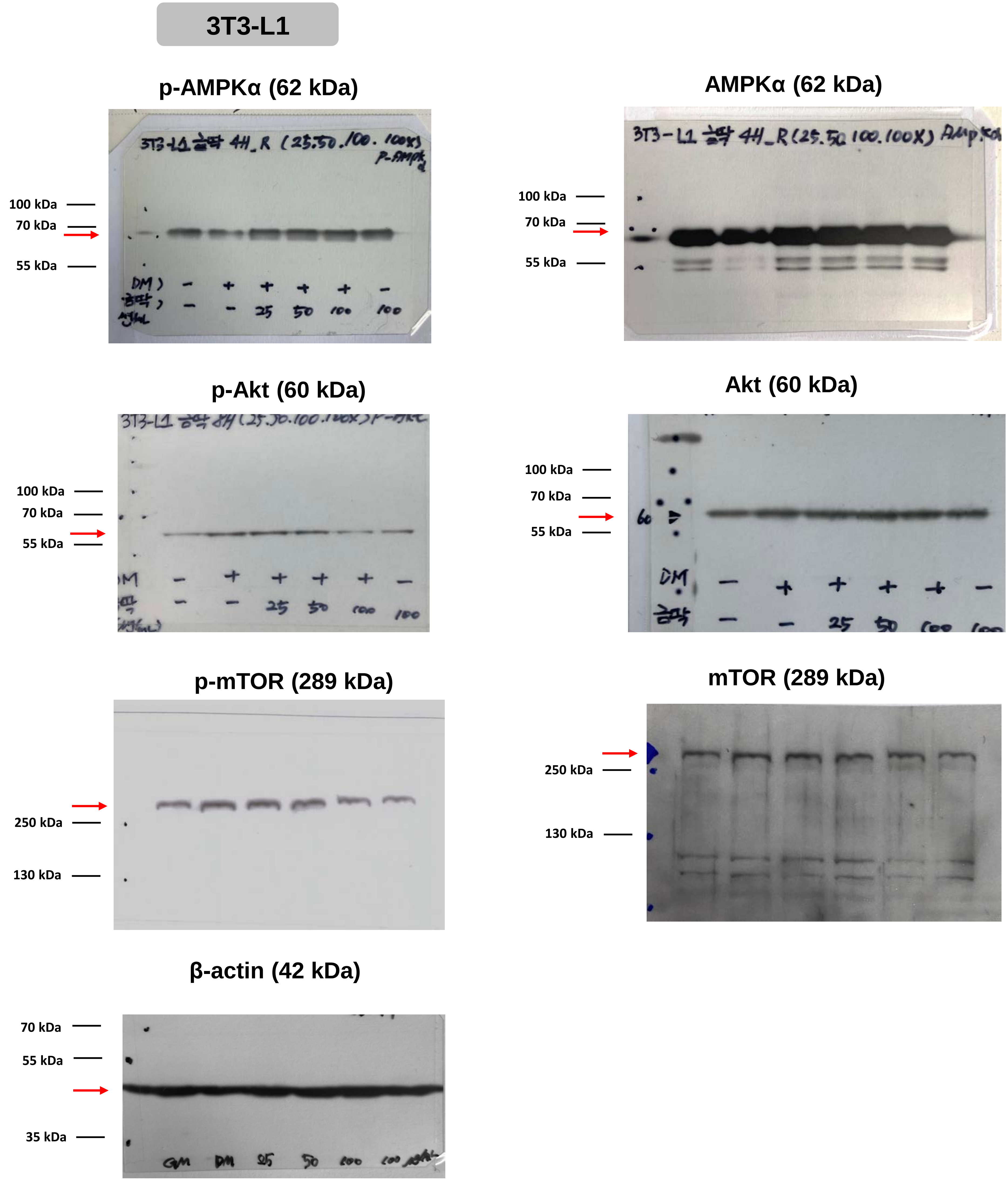
3T3-L1
p-AMPKα (62 kDa)
100 kDa
70 kDa
55 kDa
AMPKα (62 kDa)
100 kDa
70 kDa
55 kDa
p-Akt (60 kDa)
100 kDa
70 kDa
55 kDa
Akt (60 kDa)
100 kDa
70 kDa
55 kDa
p-mTOR (289 kDa)
250 kDa
130 kDa
mTOR (289 kDa)
250 kDa
130 kDa
β-actin (42 kDa)
70 kDa
55 kDa
35 kDa

Figure S3B

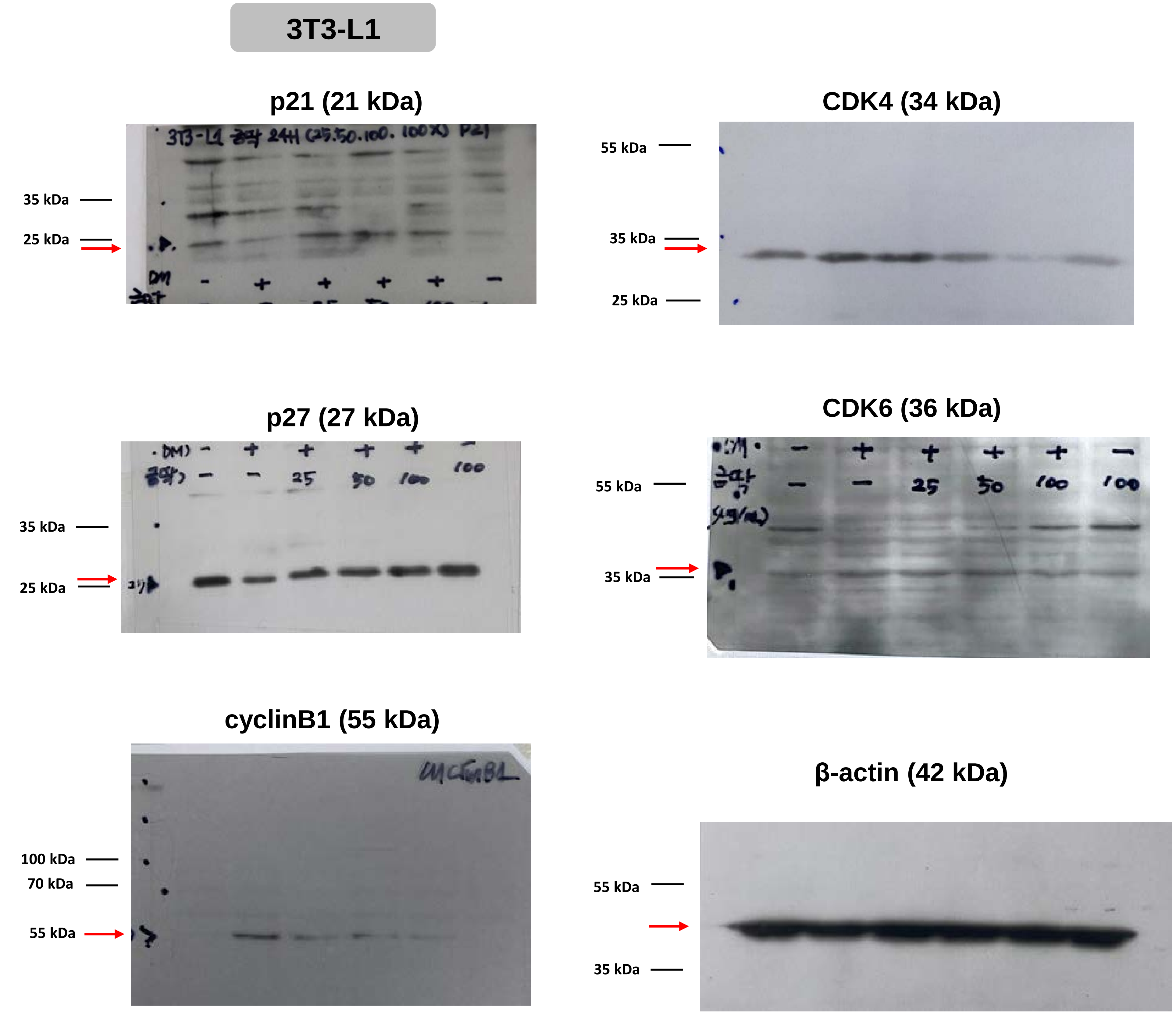
3T3-L1
p21 (21 kDa)
35 kDa
25 kDa
CDK4 (34 kDa)
55 kDa
35 kDa
25 kDa
p27 (27 kDa)
35 kDa
25 kDa
CDK6 (36 kDa)
55 kDa
35 kDa
cyclinB1 (55 kDa)
100 kDa
70 kDa
55 kDa
β-actin (42 kDa)
55 kDa
35 kDa

Figure S6C

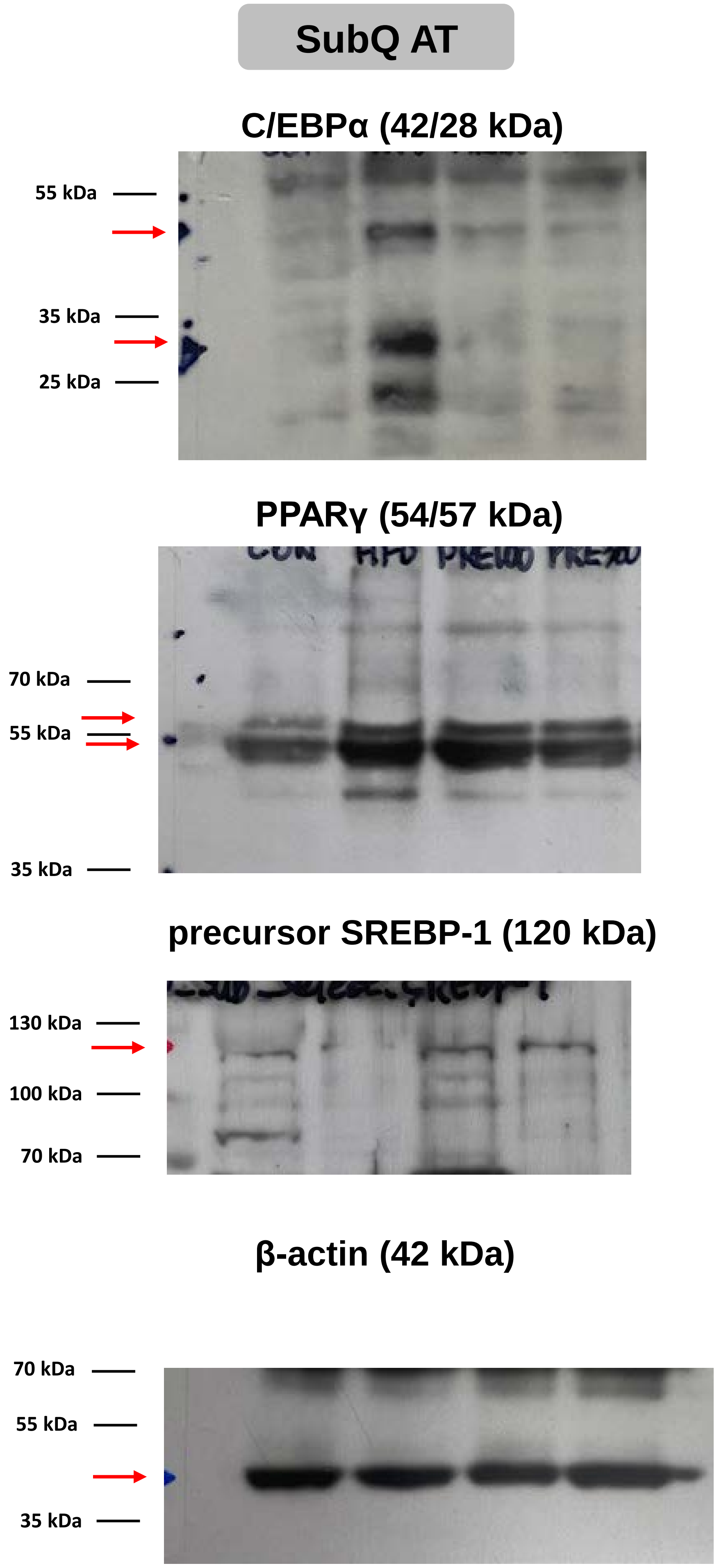
SubQ AT
C/EBPα (42/28 kDa)
55 kDa
35 kDa
25 kDa
PPARγ (54/57 kDa)
70 kDa
55 kDa
35 kDa
precursor SREBP-1 (120 kDa)
130 kDa
100 kDa
70 kDa
β-actin (42 kDa)
70 kDa
55 kDa
35 kDa

Figure S6D

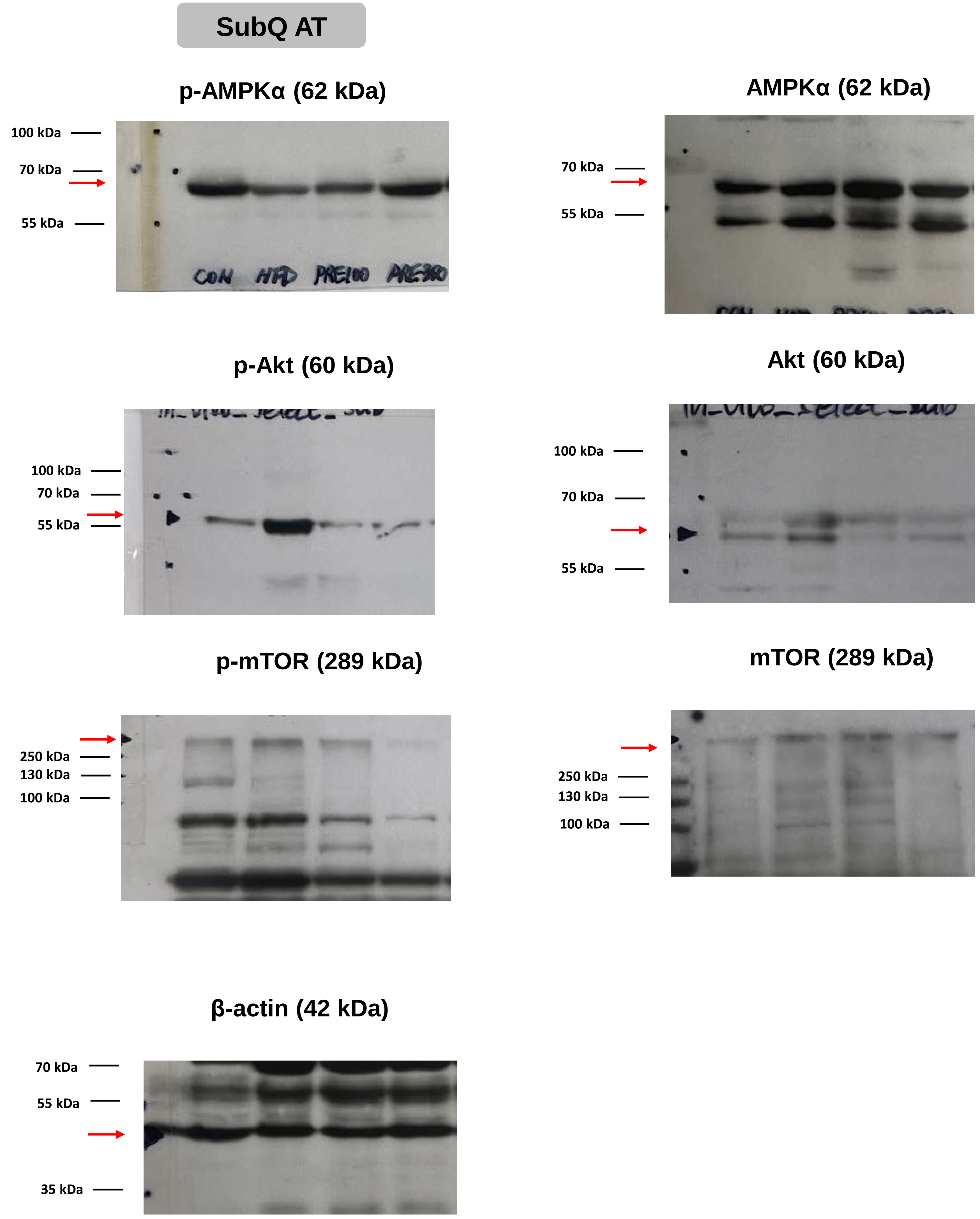
SubQ AT
p-AMPKα (62 kDa)
100 kDa
70 kDa
55 kDa
CON HFD PRE100 PRE300
AMPKα (62 kDa)
70 kDa
55 kDa
p-Akt (60 kDa)
100 kDa
70 kDa
55 kDa
Akt (60 kDa)
100 kDa
70 kDa
55 kDa
p-mTOR (289 kDa)
250 kDa
130 kDa
100 kDa
mTOR (289 kDa)
250 kDa
130 kDa
100 kDa
β-actin (42 kDa)
70 kDa
55 kDa
35 kDa

Figure S7C

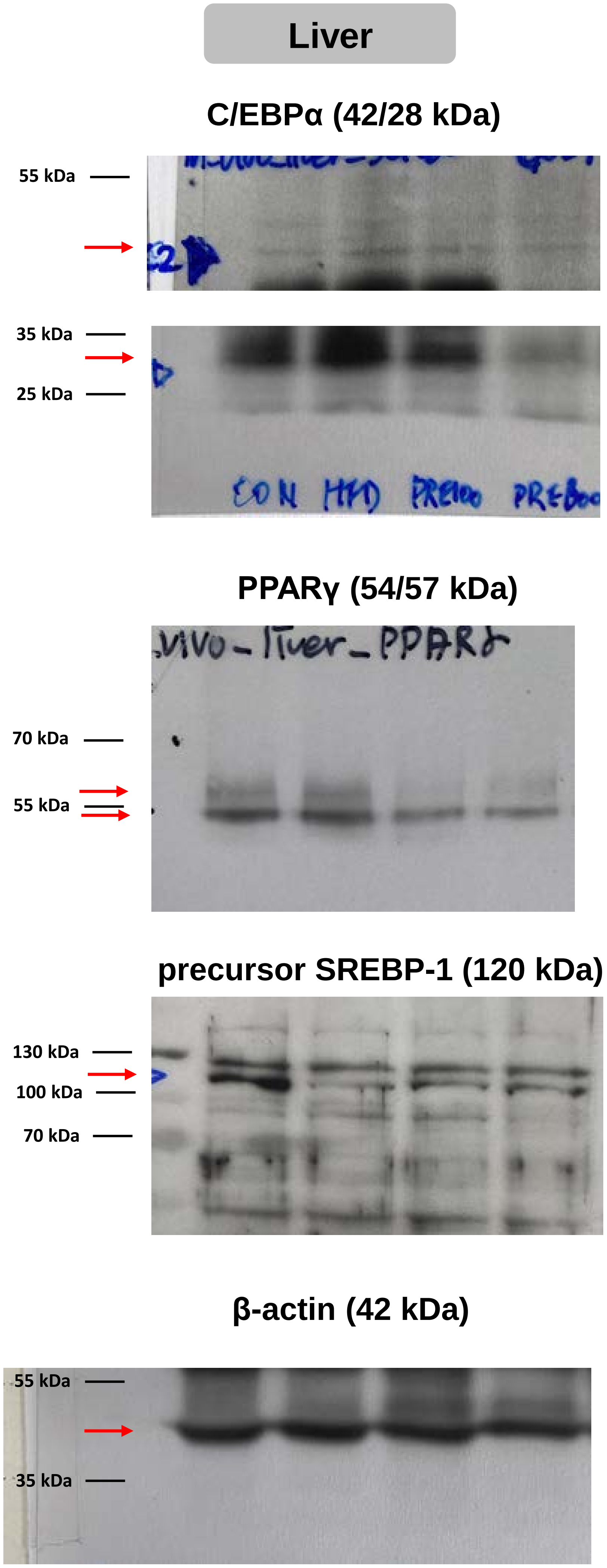

Liver
C/EBPα (42/28 kDa)
55 kDa
35 kDa
25 kDa
PPARγ (54/57 kDa)
70 kDa
55 kDa
precursor SREBP-1 (120 kDa)
130 kDa
100 kDa
70 kDa
β-actin (42 kDa)
55 kDa
35 kDa

Figure S7D

Liver

p-AMPKα (62 kDa)

70 kDa
55 kDa

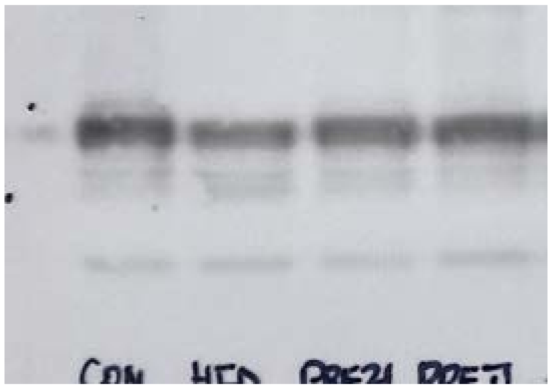

AMPKα (62 kDa)

70 kDa
55 kDa

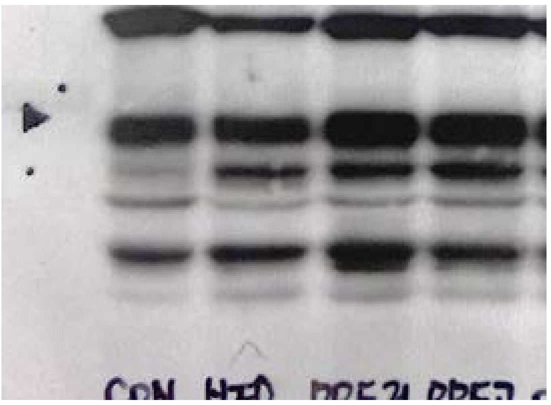

p-Akt (60 kDa)

100 kDa
70 kDa
55 kDa

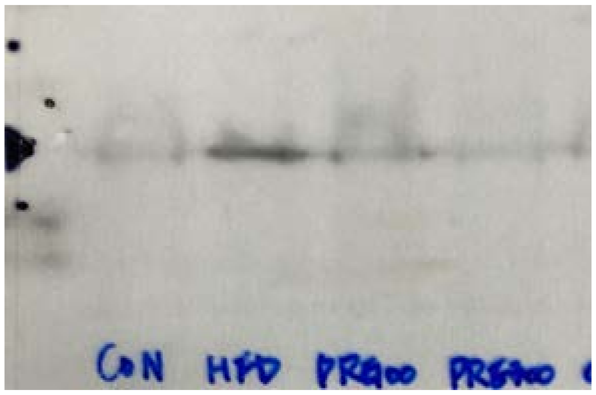

Akt (60 kDa)

70 kDa
55 kDa
35 kDa

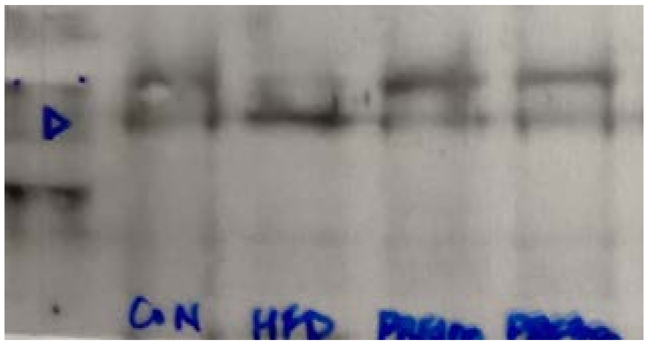

p-mTOR (289 kDa)

300 kDa
250 kDa
180 kDa

mTOR (289 kDa)

250 kDa
130 kDa
100 kDa

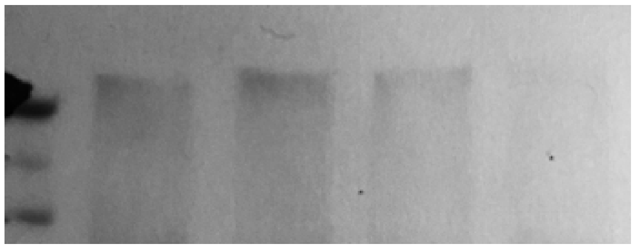

β-actin (42 kDa)

55 kDa
35 kDa

Figure S8B

Brown AT

UCP-1 (33 kDa)

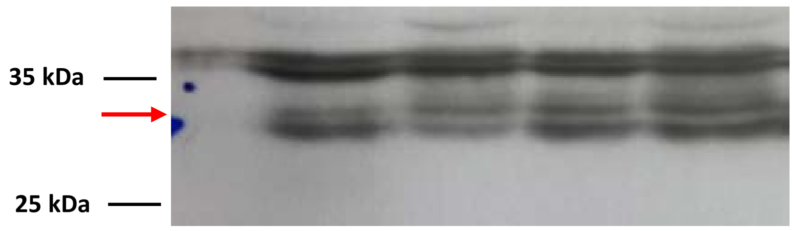

SIRT1 (120 kDa)

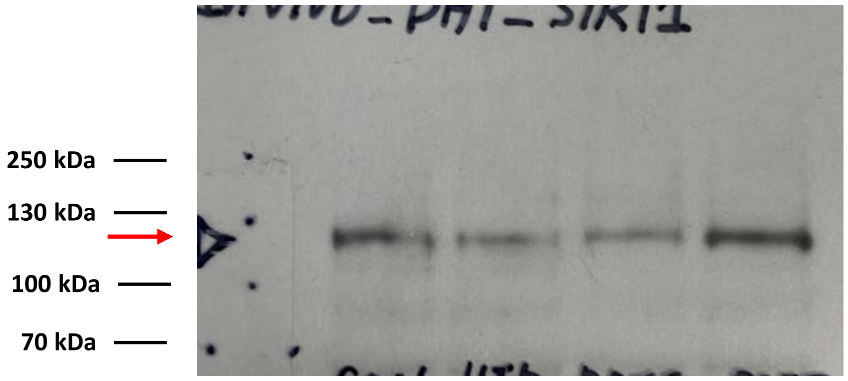

PGC-1α (130 kDa)

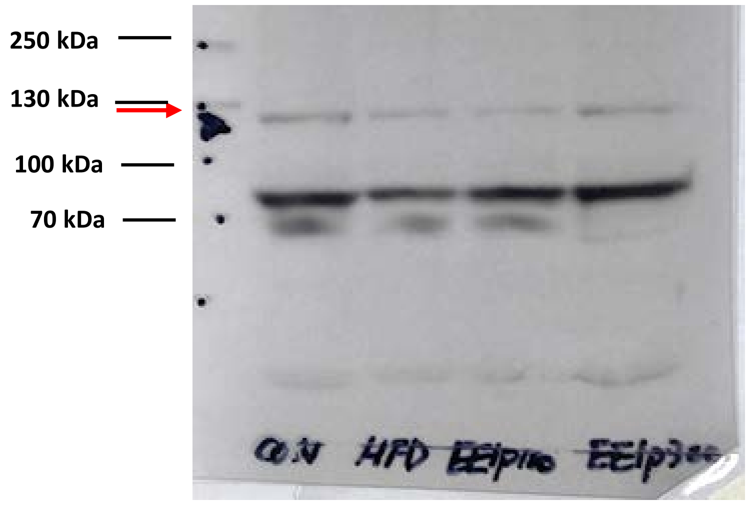

COX IV (17 kDa)

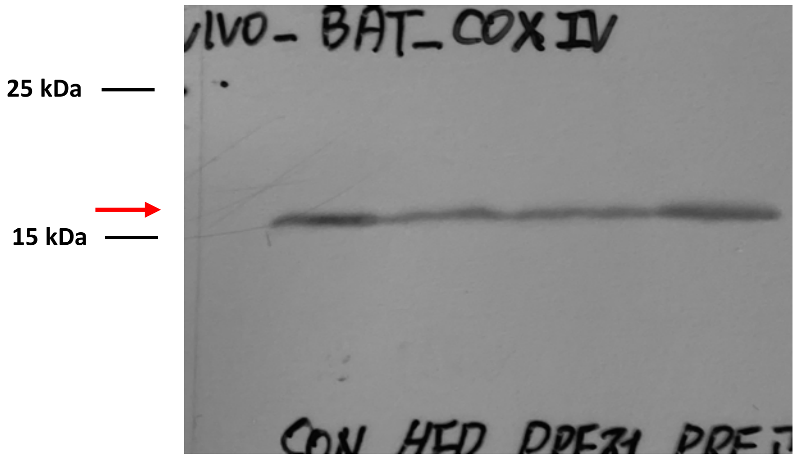

β-actin (42 kDa)

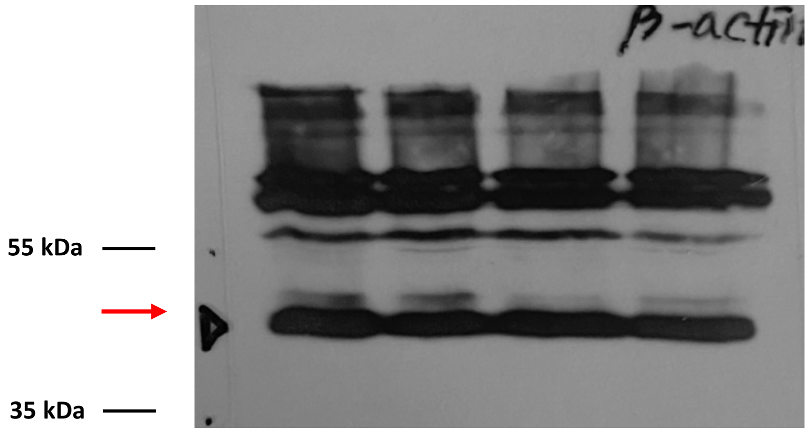

Supplement: Supplementary file 1 [file nutrients-14-03685-s001.zip › [Nutrients-1877696]supplementary material_Original blot.pdf]
