# Supplementary material for: A Botanical Mixture Consisting of Inula japonica and Potentilla chinensis Relieves Obesity via the AMPK Signaling Pathway in 3T3-L1 Adipocytes and HFD-Fed Obese Mice
Source: Nutrients. 2022 Sep 6;14(18):3685. doi: 10.3390/nu14183685 (PMC9505034; doi:10.3390/nu14183685)
Supplement: Supplementary file 1 [file nutrients-14-03685-s001.zip › [Nutrients-1877696]supplementary MnM.pdf]

nutrients

1. Materials and methods

1.1. Sample preparation

Leaves of *Inula japonica* Thunb (IJ) and *Potentilla chinensis* Ser. (PC) were collected from Sunchang-gun, Jeollabuk-do and washed and dried leaves of IJ and PC were both extracted individually with 30% EtOH (30 times w/v) at 80°C for 5 h, followed by evaporation afforded dried extract residues (35 Brix). The extract residues were sterilized for 1 h and each extract residue (IJE and PCE) was combined at a 1:1 ratio based on the solid state. Thereafter, dextrin was added to the combined mixture and homogenized so that the solid content was at 10% w/w, then the combined mixture was processed through the spray dryer to obtain a botanical preparation consisting of 30% EtOH extracts of IJ (IJE) and PC extract (PCE) complex (EEIP). Cissus extract were obtained from NutraPharmTech (Seongnam, Gyeonggi-do, Korea) and Feed was a molten artemia only for small fry and was obtained from Ace Korea.

1.2. Experiment scheme in zebrafish

After 3 days of fertilization, zebrafish fry (3 dpf) were placed in a petri dish, and then treated for 2 days of Rotifer, 6 days of Artemia, 4 days of Feed, for a total of 12 days to prepare an overfeed-induced obesity zebrafish model. Overfeed-induced obesity zebrafish model was prepared by treating Rotifer, Artemia, and feed 4 times a day, and the normal diet control group was treated twice a day. From the 11th day, IJE, PCE, EEIP and cissus extract as a positive control were treated at a concentration of 50 ug/ml in the overeating-induced obesity zebrafish model and exposed for 24 hours. After anesthetizing the prepared zebrafish fry with 0.02% tricaine, and observing the body length and belly length of the zebrafish using an optical microscope (Olympus 1×70, Olympus, Japan). Body length and belly length are measured using Focus Lite software, and the weight is measured with a precision electronic scale for analysis and graphed

1.3. Oil red O staining for assessment of lipid accumulation in zebrafish

After dosing of overfeed-induced obese zebrafish fry (larvae) was fixed overnight in 4% paraformaldehyde (PFA) (Fujifilm Wako Pure Chemical Corporation, Japan) at 4°C, washed twice with Phosphate Buffered Saline (PBS, Takara bio inc., Japan). After treatment with 80% and 100% propylene glycol (Sigma Chemical Co., USA) at room temperature for 20 minutes, respectively, the zebrafish fry was by exposing them to 0.5% oil Red (Glentham life science, UK) in 100% propylene glycol overnight at room temperature to block light. After dyeing, wash with PBS, wash with 100% and 80% propylene glycol at room temperature for 30 minutes, respectively, to wash the dye, and store in 80% propylene glycol at 4°C under an optical microscope (Olympus 1×70, Olympus, Japan) to observe the fat in the liver and measure the fat size using the Focus Lite software (Focus Co., Daejeon, Korea).

1.4. Lipid profiling of zebrafish

The lipid profile was analyzed using a cholesterol analysis kit- high-density lipoprotein (HDL) and low-density lipoprotein (LDL)/very low-density lipoprotein (VLDL)

(Abcam Inc., Cambridge MA, UK). After washing the zebrafish tissue with PBS, add 100 μl cholesterol assay buffer and homogenize with a homogenizer (bel-Art Inc., Wayne, USA). To obtain total cholesterol (T-CHO), the homogenized zebrafish tissue is centrifuged using centrifuge (Hanil Co., Incheon, Korea) at 4°C, 13,000g speed for 5-10 minutes, and the supernatant is collected. For HDL, mix the remaining solution except for T-CHO with the same amount of 2X LDL/VLDL precipitation buffer, put it in an incubator (HYSC Co., Seoul, Korea) for 10 minutes at room temperature, and then centrifuge (Hanil Co., Incheon, Korea), centrifuge the sample at room temperature for 10 minutes at a speed of 2000 g, and obtain a supernatant. The remaining precipitate was centrifuged again using a centrifuge for 10 minutes at room temperature and a speed of 2000 g, and after removing the supernatant, it was dissolved in 200 μl PBS. Obtain LDL/VLDL. 50 μl of each standard and sample were loaded on a 96 well white plate, and 50 μl of cholesterol reaction mix was added, and then placed in an incubator at 37°C under light-shielding conditions for 1 hour. Then, the absorbance was measured at 570 nm using a Microplate reader (PerkinElmer Co., Bodenseewerk, UK). The concentration measurement results for each sample are substituted into the standard curve and expressed as the average concentration (μg/mL).

1.5. Statistical analysis

Statistical analysis was performed using Sigma Plot software (Systat software Inc., USA) and all data are expressed as mean ± standard error mean. Paired t-teat and repeated ANOVA were used to check whether there was a difference between groups in each experimental condition.
